# The Flavonoid Kaempferol Mitigates Periprosthetic Osteolysis by Regulating the NLRP3 Inflammasome and Balancing Bone Metabolism

**DOI:** 10.1111/jcmm.70878

**Published:** 2025-10-06

**Authors:** Cheng Huang, Chenhui Zhang, Yongjun Luo, Lujun Guo, Yanglin Wu, Qingyan Shi, Yazhong Zhang, Chengyuan Yang, Bo Wang, Junjie Niu, Jun Lin

**Affiliations:** ^1^ Department of Orthopeadics China‐Japan Friendship Hospital Beijing China; ^2^ Department of Orthopaedics, The Fourth Affiliated Hospital of Soochow University, SuzhouDushu Lake Hospital Medical Center of Soochow University Suzhou China; ^3^ Department of Orthopaedics The Second Affiliated Hospital of XuZhou Medical University Xuzhou China; ^4^ Department of Orthopaedics, The First Affiliated Hospital of Soochow University Soochow University Suzhou China; ^5^ Department of Spinal Surgery, School of Medicine, Shanghai East Hospital Tongji University Shanghai China

**Keywords:** bone metabolism, kaempferol, NLRP3 inflammasome, osteolysis, pyroptosis

## Abstract

Total joint arthroplasty (TJA) is an effective intervention for end‐stage arthritis; however, its long‐term success is often compromised by wear particle–induced osteolysis, leading to aseptic loosening and implant failure. This study investigates the potential of kaempferol (Ka), a natural flavonoid with anti‐inflammatory properties, to alleviate osteolysis by modulating NLRP3 inflammasome activation. In a murine calvarial osteolysis model, Ka administration significantly attenuated bone loss induced by CoCrMo alloy particles. Mechanistically, Ka dose‐dependently inhibited NLRP3 inflammasome activation in macrophages, as evidenced by reduced IL‐1β secretion, decreased ASC oligomerisation and suppressed GSDMD cleavage, ultimately leading to decreased pyroptosis. These effects were found to be partially mediated via GPR109a. Furthermore, Ka markedly suppressed osteoclast differentiation and activity both in vivo and in vitro while promoting osteoblast differentiation, thereby contributing to the restoration of bone remodelling balance. Taken together, our findings suggest that Ka exerts a protective effect against wear particle–induced osteolysis by targeting the NLRP3 inflammasome and modulating osteoimmune responses, which may offer a novel therapeutic strategy to manage periprosthetic osteolysis and prolong implant longevity.

## 1 Introduction

Total joint arthroplasty (TJA) represents a highly efficacious approach in the management of end‐stage joint diseases, offering marked alleviation of pain and restoration of joint function. Over the decade spanning from 2012 to 2022, the United States witnessed a staggering total of over 3.1 million TJAs performed, with an annual trend of continued growth observed [1]. Nonetheless, these implants possess a finite lifespan and may require revision operations because of aseptic loosening, infection, periprosthetic fracture, etc. These revisions are notably complex, associated with inferior outcomes and long‐term prognoses compared to primary replacements. Consequently, this scenario imposes substantial economic and societal burdens [2, 3]. Aseptic loosening, a consequence of periprosthetic osteolysis, stands as a prominent factor influencing the longevity of TJA, with wear particles playing a pivotal role in its pathogenesis [4, 5]. Given this, there arises an imperative necessity for in‐depth investigations aimed at elucidating the underlying pathological mechanism and developing efficacious therapeutic strategies.

The inflammasome, a substantial multimeric protein complex, has a key influence on the activation of caspase‐1 and the following completion of IL‐1β maturation [6, 7, 8, 9]. Among the diverse and distinct inflammasomes, the NLRP3 inflammasome stands out as a prominent and versatile member, primarily consisting of NLRP3, ASC (apoptosis‐associated speck‐like protein containing CARD) and pro‐caspase‐1 [10]. NLRP3, belonging to the family of NLRs (Nod‐like receptors), possesses the remarkable ability to discern a range of danger‐ or pathogen‐associated molecular patterns (DAMPs or PAMPs) [11]. ASC, a pivotal protein, facilitates the recruitment of pro‐caspase‐1 and its subsequent connection to NLRP3 during the inflammasome assembly process [12]. The activation of the NLRP3 inflammasome necessitates a dual‐step mechanism. Initially, Toll‐like receptors (TLRs) are indispensable for generating a priming signal [10]. TLR agonists activate NF‐κB, which subsequently drives the synthesis of pro‐IL‐1β and NLRP3. Subsequently, activation of the NLRP3 inflammasome occurs as a reaction to signals originating from DAMPs or PAMPs, including but not limited to nigericin, urate, ATP, amyloids, silica and wear particles [13, 14, 15, 16]. Furthermore, Gasdermin D (GSDMD), a protein comprised of an N‐terminus and C‐terminus, is recruited by the active NLRP3 inflammasome. Upon caspase‐1 cleavage, GSDMD‐N (the N‐terminus) dissociates from GSDMD, forming transmembrane pores that regulate IL‐1β secretion. This cascade ultimately culminates in cellular lysis and pyroptosis [17].

Excessive inflammasome activation is confirmed to play a pivotal role in regulating periprosthetic osteolysis stimulated by wear particles [16, 18]. These wear particles surrounding the implant elicit a robust inflammatory cascade, ultimately contributing to the loosening of the joint prosthesis. Mechanistically, wear particles act as triggers for the activation of the NLRP3 inflammasome, leading to the secretion of cleaved IL‐1β [18]. IL‐1β, emanating from macrophages, can recruit osteoclast precursors and then promote osteoclast formation [19]. Furthermore, IL‐1β exerts a detrimental influence on osteogenic differentiation, further disrupting the delicate equilibrium between bone resorption and bone formation [20]. Consequently, the progressive imbalance between these two processes precipitates the transition of periprosthetic osteolysis into aseptic loosening. Therefore, the regulation of the NLRP3 inflammasome and bone metabolic pathways offers promising directions for therapeutic strategies against osteolysis.

Kaempferol (Ka) is a natural polyphenol in the group of flavonoids and is widely present in a variety of dietary sources such as vegetables, fruits and teas, especially enriched in many medicinal herbs [21, 22, 23]. It has been reported to exert various physiological effects, particularly notable for its potent anti‐inflammatory and antioxidant properties [24, 25]. Recent evidence indicates that Ka can mitigate particle‐induced osteolysis, primarily by inhibiting osteoclast differentiation and function [26] or enhancing osteogenesis [27]. Nevertheless, upon exposure to wear particles, macrophages are the first to phagocytose and become activated, subsequently promoting bone resorption pathways while suppressing bone formation [28], playing a critical role in the generation of wear particle–induced osteolysis [29]. Despite these insights, the regulatory effects of Ka on macrophage‐mediated immune responses in osteolysis remain unexplored.

**FIGURE 1:**
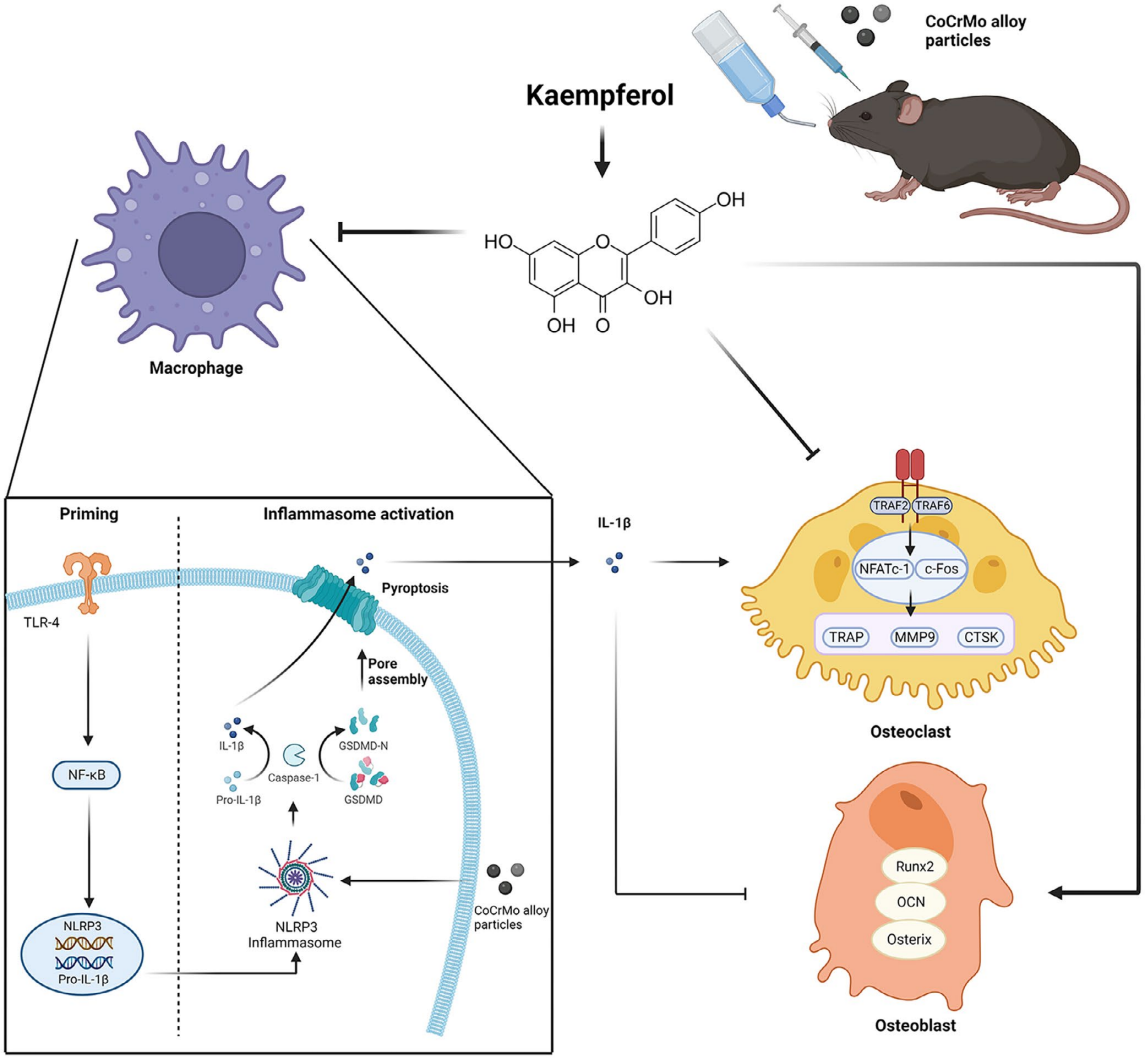
Schematic illustrations of the flavonoid kaempferol mitigates periprosthetic osteolysis by regulating the NLRP3 inflammasome and balancing bone metabolism.

In this study, we initially delved into the impact of Ka on mitigating the osteolytic effects induced by CoCrMo alloy particles in mice, specifically examining its inhibitory effect on NLRP3 inflammasome activation. Furthermore, we expanded our inquiry to evaluate the impact of Ka on the processes of osteoblast and osteoclast differentiation, elucidating the role of IL‐1β inhibition in these processes. Consequently, our findings indicate that Ka regulates the equilibrium between osteogenesis and osteolysis by suppressing NLRP3 inflammasome‐driven macrophage pyroptosis and the release of IL‐1β, ultimately preventing wear particle–induced osteolysis (Figure 1).

## 2 Methods

### 2.1 Materials and Reagents

Ka and PMA were procured from MCE (USA), whereas LPS was obtained from Sigma (USA). The cell lines, THP‐1 and MC3T3‐E1, were acquired from Procell (Wuhan, China). Additionally, M‐CSF was sourced from R&D Systems (USA). For molecular biology procedures, we utilised RIPA lysis buffer, BCA assay kit, Quickblock blocking buffer and polyvinylidene difluoride membranes, all of which were purchased from Beyotime (China). For the assessment of bone resorption, the Tartrate‐resistant Acid Phosphatase (TRAP) kit was sourced from BZ Biotechnology (China). Lastly, for RNA extraction, we employed the Simple P Total RNA Extraction Kit, sourced from Hangzhou Bioer Technology Co. Ltd.

For antibodies, Actin was obtained from Beyotime (China); Runx2, ASC and caspase‐1 were acquired through Cell Signaling Technology (USA); c‐Fos, NFATc‐1, IL‐1β, GSDMD and NLRP3 were from Abcam (USA); TRAP, CTSK and GPR109a were from Santa Cruz (USA); TRAF2, TRAF6 and MMP9 were from Proteintech (China); osterix and OCN were from Affinity Biosciences (China).

### 2.2 Mouse Osteolysis Models

The mice, procured from Suzhou Healthytech Bio‐pharmaceutical Co. Ltd., underwent an experimental paradigm designed to induce wear particle‐mediated osteolysis. Specifically, 8‐week‐old C57BL/6 mice underwent the surgical placement of CoCrMo alloy particles onto the calvarial surface, followed by a 2‐week observation period. Prior to the procedure, mice were anaesthetised and their scalps shaved. Subsequently, the surgical site was thoroughly disinfected using an iodophor solution. Following this, a precise 10 mm sagittal incision was meticulously crafted to expose the calvaria, and the periosteum was delicately scraped away. Subsequently, 20 mg of CoCrMo alloy particles, suspended in 40 μL of phosphate‐buffered saline (PBS), were uniformly dispersed over the calvarial surface. Once the particles were evenly distributed, the incision was meticulously closed with sutures and subsequently sterilised using iodophor.

The experimental design entailed the systematic allocation of mice into four groups, which comprised the control group (designated as sham), a group subjected to exposure to CoCrMo particles (labelled as CoCrMo), a group that underwent intervention with low concentrations of Ka (designated as low‐Ka, at a dosage of 25 mg/kg) and a group that received intervention with high concentrations of Ka (designated as high‐Ka, at a dosage of 100 mg/kg). After a consistent 14‐day recovery period post‐operatively, the mice were subjected to humane euthanasia in preparation for micro‐computed tomography (micro‐CT) scanning and subsequent histological assessment.

### 2.3 Micro‐CT Analysis

Details are provided in Data S1.

### 2.4 SEM Image of Particles

SEM (scanning electron microscopy) image of CoCrMo particles was conducted by CeshiGo. Co. Ltd. (Nanjing, China).

### 2.5 Cell Cultures

Details are provided in Data S1.

### 2.6 Enzyme‐Linked Immunosorbent Assay

Cell supernatants were extracted for the analysis of IL‐1β and IL‐18 by the ELISA kit (MultiScience, Hangzhou, China) under the guidance of the manufacturer's instruction.

### 2.7 Analysis of ASC Oligomerisation

Details are provided in Data S1.

### 2.8 Alkaline Phosphatase (ALP) Staining

After 1‐week incubation in osteogenic medium, MC3T3‐E1 cells were subjected to alkaline phosphatase (ALP) staining utilising the BCIP/NBT kit from Beyotime, strictly adhering to the manufacturer's protocol.

### 2.9 Alizarin Red S (ARS) Staining

The MC3T3‐E1 cells were allowed to grow in an osteogenic medium over a period of 20 days. Subsequently, the cells underwent fixation using a 4% paraformaldehyde solution for a duration of 20 min to maintain their structural integrity. Subsequently, the cells were stained with ARS solution for approximately 30 min to visualise mineralised nodules. After three rinses with ddH_2_O, images of the mineralisation process were captured.

### 2.10 qRT‐PCR

Details are provided in Data S1 and described in the previous report [30].

### 2.11 Western Blotting

Details are provided in Data S1.

### 2.12 TRAP, Immunohistochemistry and Immunofluorescence Staining

The mouse calvarium was treated with a neutral decalcifying solution for approximately 4 weeks before being embedded in paraffin. H&E staining was conducted according to the manufacturer's instructions. The TRAP kit was utilised for staining the calvarium slices and osteoclasts.

For immunohistochemistry, calvarial slices were incubated with caspase‐1, IL‐1β and NLRP3 for 12 h. On the subsequent day, the tissue sections were thoroughly rinsed three times. Following this, they were incubated with secondary antibodies at ambient temperature for a period of 1 h. Finally, the calvarial slices were stained with 100 μL of DAB for 3–5 min and counterstained with haematoxylin.

For immunofluorescence, mature osteoclasts and differentiated osteoblasts were first fixed with a 4% paraformaldehyde solution for 20 min. Following this, the cells were subjected to overnight incubation with the respective primary antibodies at a temperature of 4°C. After three rounds of washing, a secondary fluorescent antibody was applied and incubated for 1 h. The cell nuclei were counterstained with DAPI for 3 min to visualise nuclear morphology.

In the Calcein/PI staining procedure, after stimulation with LPS, cells were treated with or without Ka and CoCrMo alloy particles. Calcein‐AM and PI were then used for staining in each well for 30 min at 37°C.

### 2.13 Statistical Analysis

Results are expressed as the average ± standard deviation (SD). The statistical evaluation was performed using Sigmaplot software, version 14.0 (Systat Software, San Jose, CA, USA). To assess the statistical significance of differences both between and within experimental groups, a one‐way or two‐way analysis of variance (ANOVA) was applied. A *p*‐value of less than 0.05 was considered to indicate statistical significance.

## 3 Results

### 3.1 Characterisation of CoCrMo Alloy Particles

For several decades, CoCrMo alloys have been extensively utilised in load‐bearing implants, notably in hip and knee joint prosthetics, owing to their superior mechanical properties and notably low wear rates. Consequently, to investigate the morphology of CoCrMo particles, scanning electron microscopy (SEM) was employed. As depicted in Figure S1, the particles exhibited an irregular spherical shape and lacked uniformity in size, with diameters spanning from 0.01 to 10 μm and an average diameter of 1.65 μm. This size range is conducive to cellular uptake and has been observed in the vicinity of implants [31]. Consequently, CoCrMo alloy particles within this size spectrum were selected for further investigation in our study.

### 3.2 Kaempferol (Ka) Inhibits Osteolysis In Vivo

**FIGURE 2:**
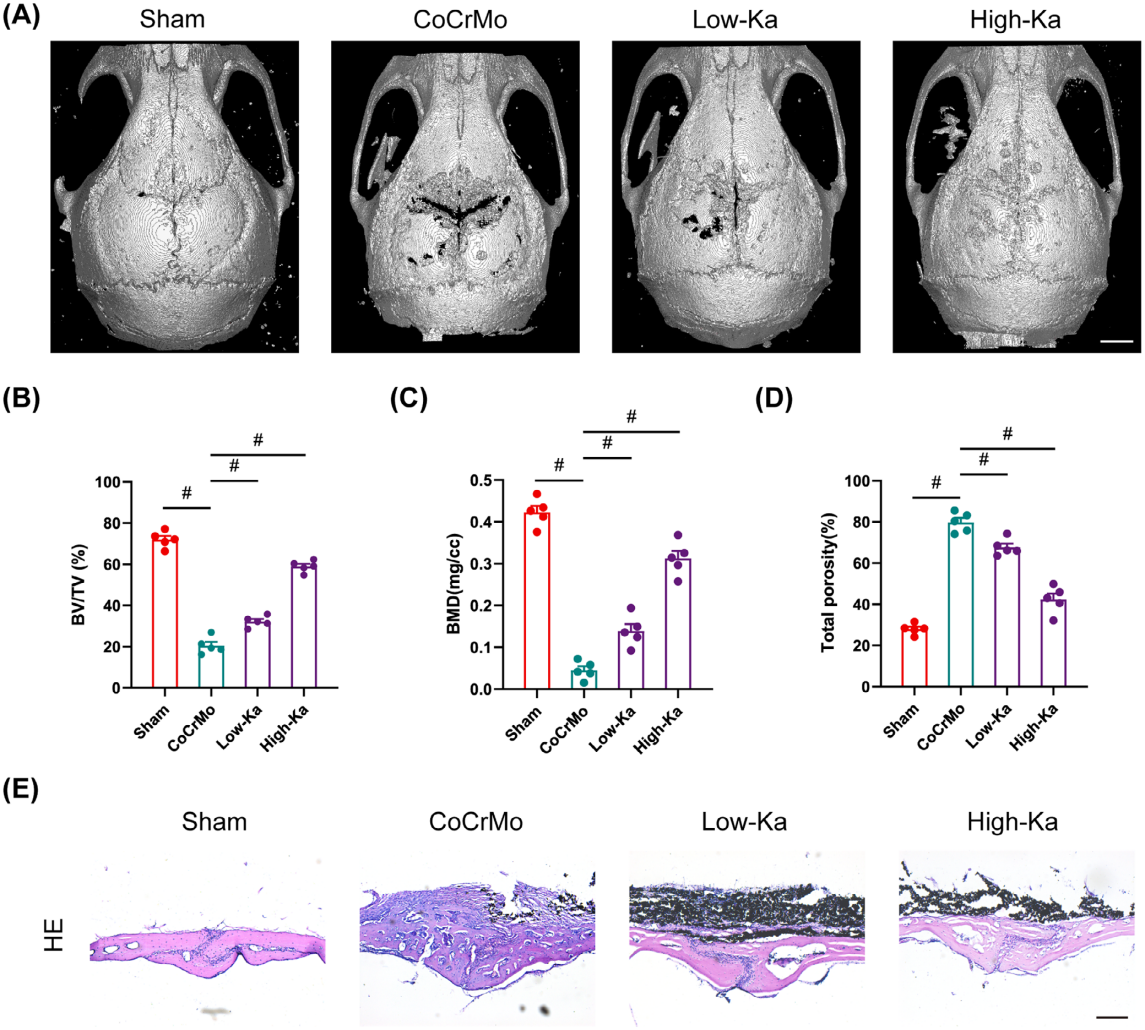
Ka inhibits osteolysis stimulated by CoCrMo in vivo. (A) General 3D rebuilding images of mouse calvarium. Scale bar was 5 mm. (B–D) Quantitation of related bone parameters, including BV/TV (%), BMD (mg/cc) and total porosity (%). (E) General H&E staining images of mouse calvarium slices. Scale bar was 200 μm. All data represent mean ± SEM. ^#^ *p* < 0.001.

To investigate the in vivo effects of Ka on osteolysis induced by CoCrMo alloy particles, an osteolysis model was established in C57BL/6 mice. Following surgery, mice were administered Ka or left untreated for a duration of 2 weeks. Subsequently, the animals were euthanised for Micro‐CT analysis. The 3D reconstruction images showed profound alterations in the microarchitecture of the calvaria, characterised by pronounced bone loss, indicative of an elevated degree of calvarial osteolysis in the presence of CoCrMo alloy particles (Figure 2A). In contrast to the sham group, key bone metrics, including bone mineral density (BMD) and the ratio of bone volume to total volume (BV/TV), were evaluated and were markedly reduced in the CoCrMo particle treatment group, whereas total porosity exhibited a significant decrease (Figure 2B–D). Notably, both the 3D images and bone parameters demonstrated a distinct protective effect of Ka against osteolysis stimulated by CoCrMo particles (Figure 2A–D). Furthermore, histological examination corroborated these findings, revealing reduced bone erosion in mice treated with Ka. Haematoxylin and eosin (HE) staining highlighted severe tissue damage in the calvaria of the CoCrMo treatment group, in contrast with the sham group. However, Ka treatment was associated with decreased inflammation and osteolysis, as evidenced by the histological sections (Figure 2E). Collectively, these results underscore the potential of Ka to suppress wear particle–induced osteolysis in vivo.

### 3.3 Ka Represses the NLRP3 Inflammasome Activation

**FIGURE 3:**
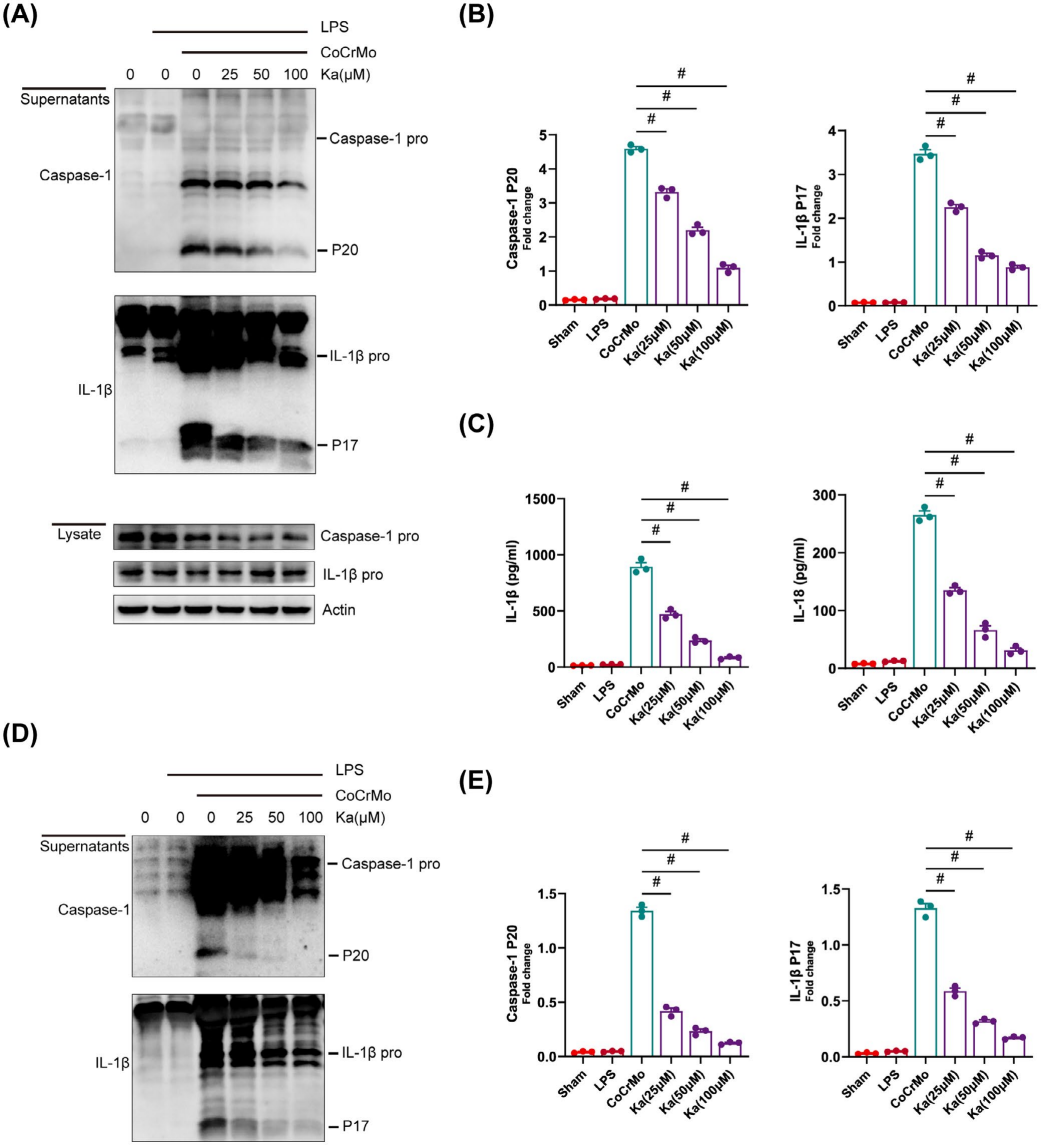
The inhibitory effect of Ka on inflammasome activation stimulated by CoCrMo. (A–C) LPS‐primed BMDMs were stimulated by CoCrMo particles and intervened with different doses of Ka, the cell lysates and supernatants were collected for ELISA and western blotting. (A) Detection of cleaved IL‐1β and caspase‐1 (P17 and P20) by immunoblotting. (B) Quantification of active IL‐1β and caspase‐1. (C) Detection of IL‐1β and IL‐18 secretion by ELISA. (D, E) NLRP3 inflammasome was activated in THP‐1 cells with or without Ka intervention. (D) Immunoblotting analysis of active IL‐1β and caspase‐1 in THP‐1 culture supernatants. (E) Quantification of cleaved IL‐1β and caspase‐1 from THP‐1. All data represent mean ± SEM. ^#^ *p* < 0.001.

Recent research studies have extensively documented that NLRP3 inflammasome activation is crucial for the development of osteolysis around prosthetic implants, a condition often initiated by wear particles from the implant [32]. Given the prevalent utilisation of CoCrMo alloys in load‐bearing implants, we explored the inhibitory potential of Ka regarding the initiation of the NLRP3 inflammasome cascade, utilising CoCrMo alloy particles as a representative model for particulate stimulation. LPS‐primed bone marrow‐derived macrophages (BMDMs) were challenged with CoCrMo particles, either alone or in combination with Ka treatment, followed by the collection of cell lysates and supernatants for western blotting analysis. Our findings revealed a significant suppression of NLRP3 protein expression in Ka‐treated cells (Figure S2). Furthermore, we examined the production of mature forms of pro‐IL‐1β and pro‐caspase‐1, demonstrating a dose‐dependent decrease in the generation of mature IL‐1β and caspase‐1 (P17 and P20) fragments upon Ka treatment, as evidenced in Figure 3A,B. Concordantly, ELISA‐based assessments revealed a Ka‐mediated blockage in the secretion of mature IL‐1β and IL‐18 (Figure 3C). These inhibitory effects were replicated in THP‐1 macrophages (human macrophages), as shown in Figure 3D,E.

To validate the physiological relevance of these findings, we extended our investigation to an in vivo setting, employing immunohistochemical staining to evaluate the expression of proteins associated with NLRP3 inflammasome (caspase‐1, IL‐1β, NLRP3) in mouse calvaria. Comprehensive imaging and quantitative analyses confirmed that Ka's suppressive effect on inflammasome activation persisted in vivo (Figure S3). Altogether, Ka could effectively attenuate the wear particle–induced activation of the NLRP3 inflammasome.

### 3.4 Ka Blocks the ASC Assembly and Speck Formation in Macrophages

**FIGURE 4:**
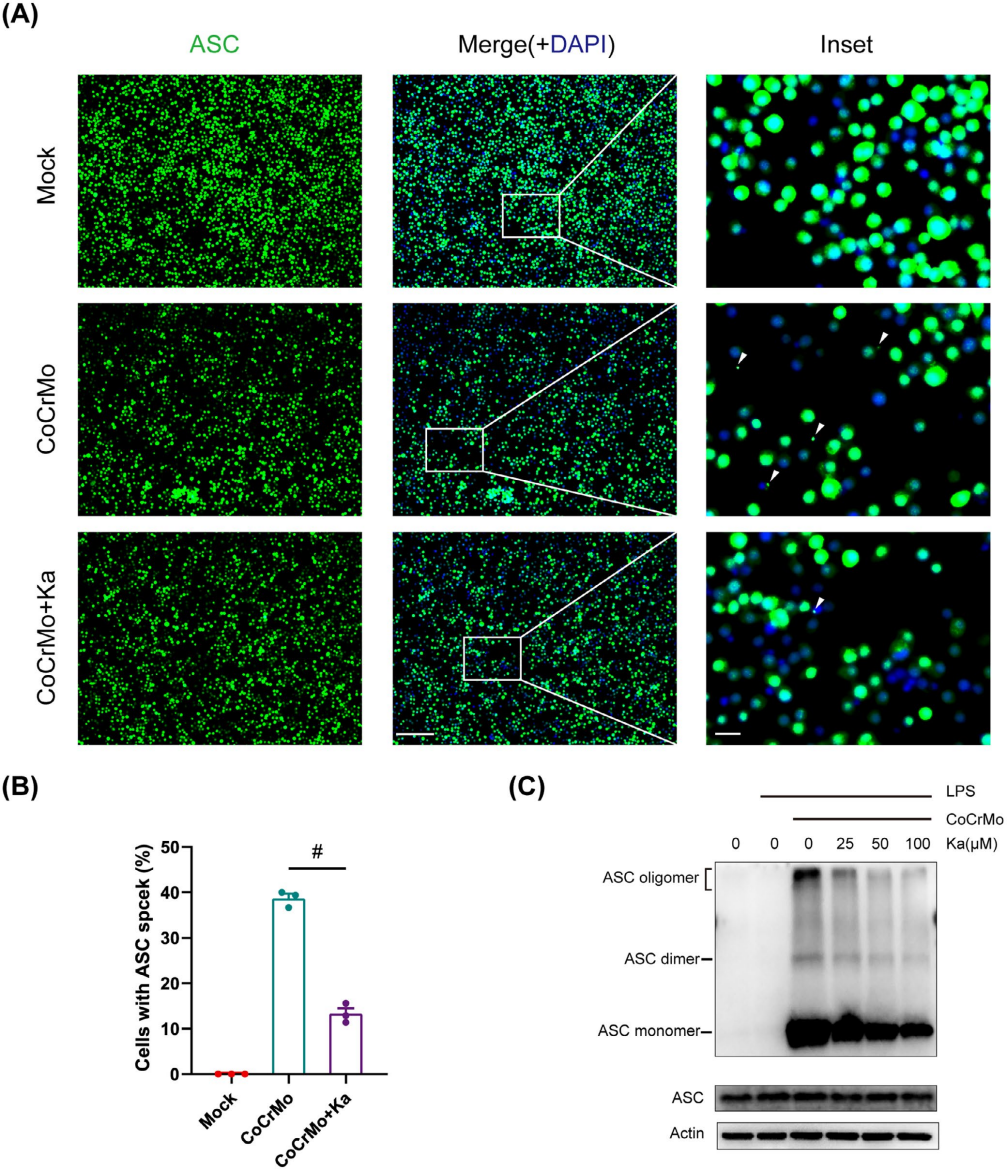
Ka blocks the ASC assembly and speck formation. (A–C) LPS‐primed BMDMs were stimulated by CoCrMo particles and intervened with different doses of Ka. (A) Immunofluorescence analysis of ASC specks in BMDMs. The arrows represent ASC specks (green). Scale bars were 200 μm (panel) and 20 μm (inset). (B) Quantification of macrophages containing ASC specks. (C) Analysis of ASC oligomerisation by immunoblotting. All data represent mean ± SEM. ^#^ *p* < 0.001.

The oligomerisation of the adaptor protein ASC serves as a pivotal indicator of inflammasome activation [33]. Upon activation of the inflammasome, ASC is recruited to form a unique, large aggregate protein complex, termed the ASC speck. Immunofluorescence assays revealed a notable upregulation in the assembly of ASC specks following stimulation with CoCrMo particles. However, upon intervention with Ka, a significant decrease in the abundance of ASC specks was observed (Figure 4A,B). To further investigate the influence of Ka on ASC oligomerisation, we employed chemical cross‐linking techniques. The subsequent Western Blot analysis confirmed that Ka exerts a dose‐dependent suppressive effect on ASC oligomerisation (Figure 4C). As a whole, these results underscore the capability of Ka to modulate NLRP3 inflammasome activation by regulating ASC assembly and speckle formation.

### 3.5 Ka Can Inhibit GSDMD Cleavage to Block Pyroptosis

**FIGURE 5:**
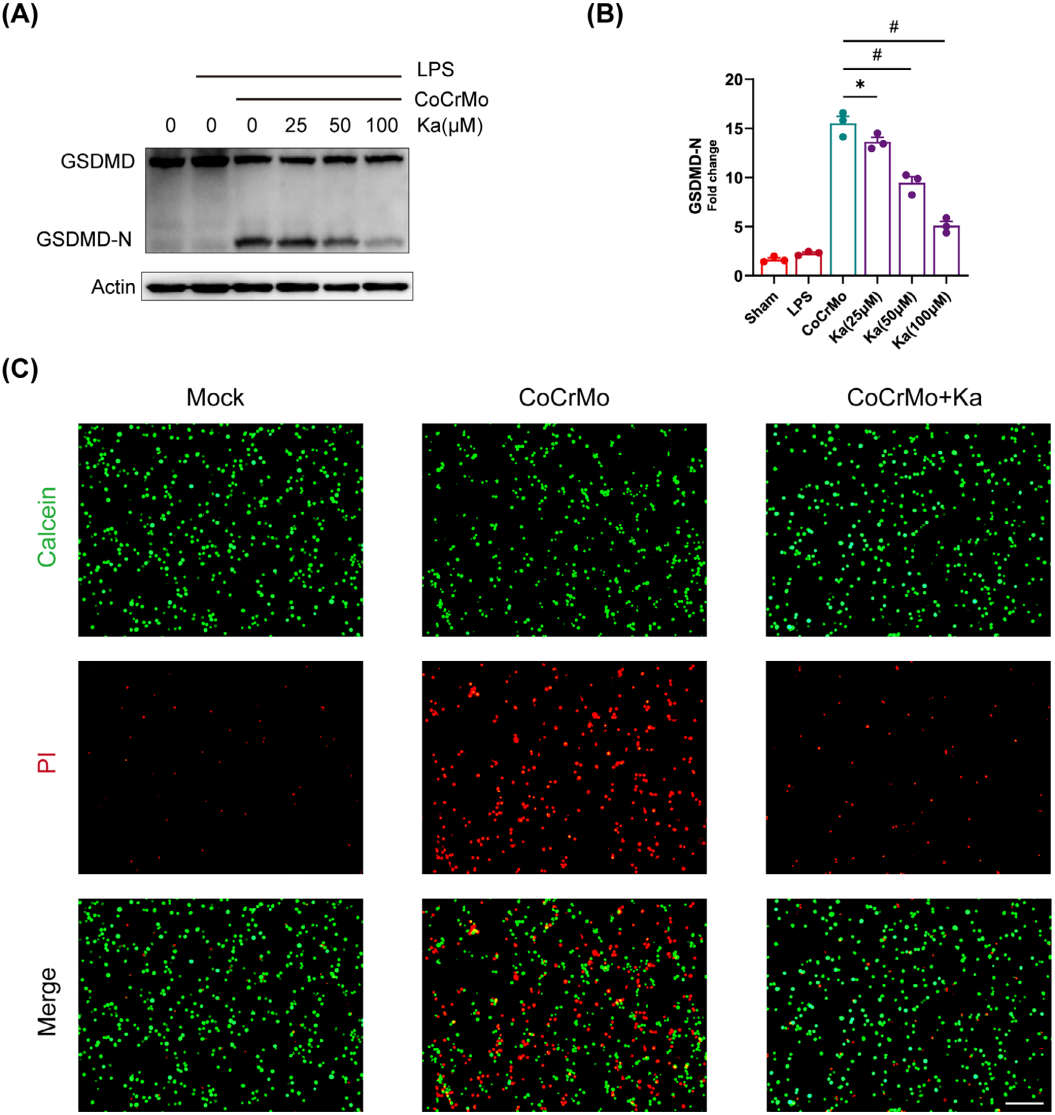
Ka restrains GSDMD cleavage to block pyroptosis. (A–C) LPS‐primed BMDMs were stimulated by CoCrMo particles and intervened with different doses of Ka. (A) Immunoblotting analysis of GSDMD; GSDMD: Gasdermin D, GSDMD‐N: GSDMD N‐terminus. (B) Quantification of GSDMD‐N. (C) General images of Calcein/PI staining. Scale bar was 200 μm. All data represent mean ± SEM. *p < 0.05. ^#^ *p* < 0.001.

The GSDMD‐N fragment, resulting from the proteolytic cleavage of GSDMD, is known as the pyroptosis executioner [34]. Consequently, we embarked on an investigation to determine whether Ka could impede the cleavage of GSDMD and subsequently attenuate pyroptosis. Utilising western blot analysis, a dose‐responsive suppression of GSDMD‐N production by Ka was noted, in contrast to the control cohort that was treated with CoCrMo particles (Figure 5A,B). Furthermore, to quantify the extent of cell death arising from inflammasome activation, we conducted Calcein/PI staining. The result revealed that Ka significantly mitigated the pyroptotic demise elicited by CoCrMo particles (Figure 5C). Consequently, Ka exerts its inhibitory effect on pyroptosis by blocking the cleavage of GSDMD.

### 3.6 The Effects of Ka on Inflammasome Activation Are Not Completely Dependent on GPR109a

**FIGURE 6:**
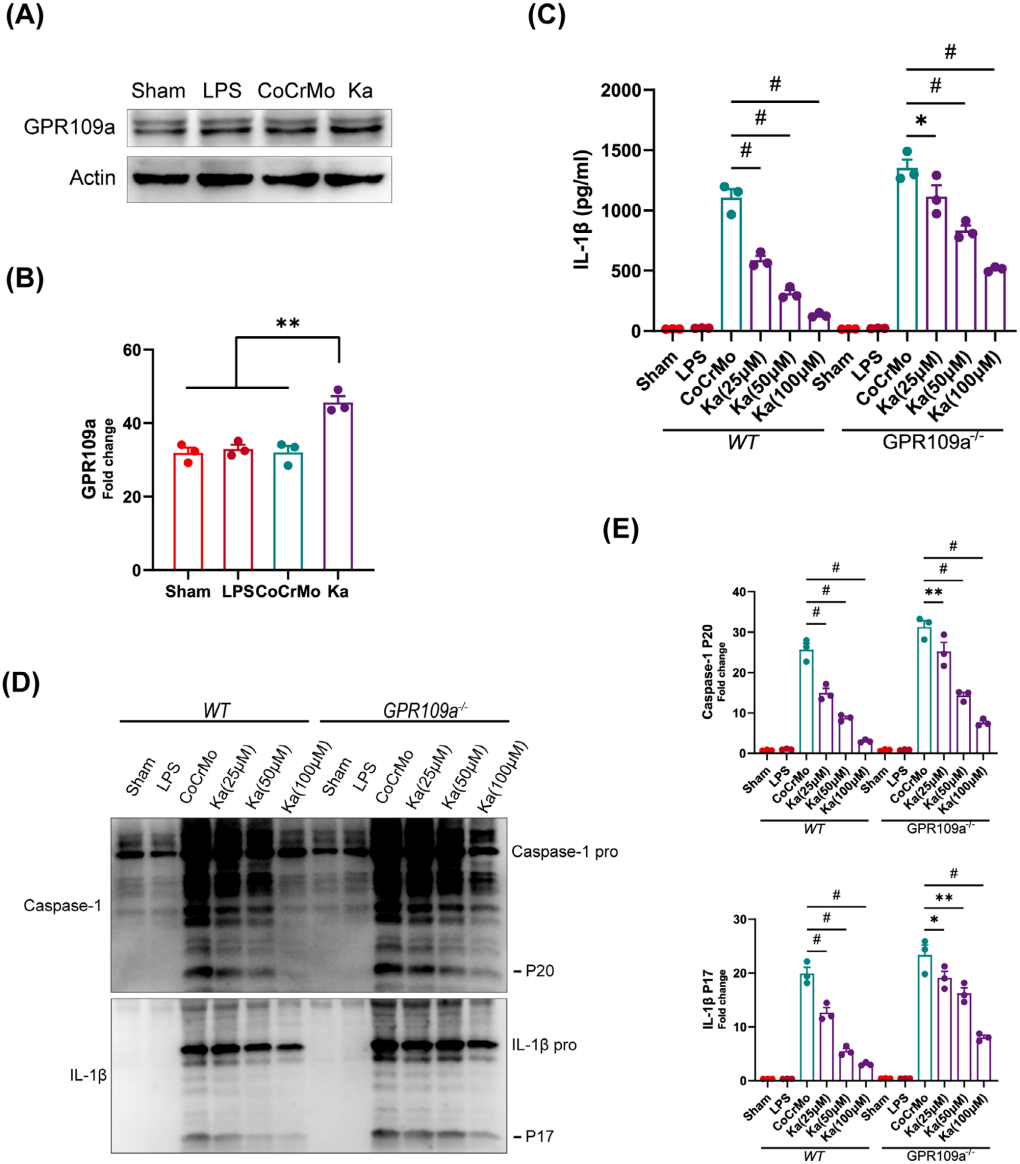
The effects of Ka on NLRP3 inflammasome do not depend on the GPR109a receptor. (A, B) BMDMs were stimulated by CoCrMo particles and treated with or without Ka. (A) Analysis of GPR109a expression in BMDMs by immunoblotting. (B) Quantification of GPR109a. (C–E) BMDMs from wild‐type and GPR109a^−/−^ mice were respectively intervened with different doses of Ka under the stimulation of CoCrMo particles. (C) Detection of IL‐1β secretion by ELISA. (D) Immunoblotting images and (E) quantification of cleaved IL‐1β and caspase‐1 from two types of mice. All data represent mean ± SEM. *p < 0.05. **p < 0.01. ^#^ *p* < 0.001.

GPR109a, a receptor recognised for its affinity to butyrate, has been reported to be modulated by Ka [35]. Prior investigations have established that butyrate exerts its inhibitory influence on inflammasome activation by the regulation of GPR109a [19]. Consequently, we embarked on a study to determine if the effects of Ka on the NLRP3 inflammasome were contingent upon the GPR109a receptor. To this end, BMDMs were subjected to stimulation with CoCrMo particles, with and without Ka treatment, followed by immunoblotting analysis. As depicted in Figure 6A,B, an elevated expression of GPR109a was observed in BMDMs treated with Ka compared to untreated controls. Subsequently, BMDMs with either sufficient or deficient GPR109a levels were treated with varying doses of Ka under CoCrMo stimulation. ELISA analysis revealed that Ka significantly reduced the release of IL‐1β in a dosage‐dependent fashion in normal BMDMs. Nevertheless, the inhibitory impact was significantly diminished in GPR109a‐deficient BMDMs (Figure 6C). Furthermore, assessment of cleaved IL‐1β and caspase‐1 levels in cell culture supernatants yielded congruent findings, reinforcing our ELISA results (Figure 6D,E).

To elucidate the in vivo effects of GPR109a on modulating the inflammatory response, we utilised both wild‐type and GPR109a knockout mice as experimental models to induce osteolysis via CoCrMo particle administration. These mice were subsequently treated with or without Ka, a pharmacological agent, for a duration of 2 weeks, followed by euthanasia for comprehensive immunohistochemical evaluation. Our findings revealed a robust and consistent suppression of NLRP3 protein expression in wild‐type mice upon Ka intervention. Intriguingly, while Ka retained a degree of therapeutic efficacy in GPR109a knockout mice, its potency was notably diminished (see Figure S4A,D for details). To delve deeper into the mechanistic underpinnings, we scrutinised the expression patterns of IL‐1β and caspase‐1 in calvarial tissue sections derived from both mouse strains. Representative microscopic images (Figure S4B,C) and quantitative assessments of positive cell counts underscored the attenuated therapeutic response to Ka in GPR109a‐deficient mice compared to their wild‐type counterparts (Figure S4E,F). Collectively, these data indicate that Ka delivers a modulatory impact on the NLRP3 inflammasome, potentially via the GPR109a receptor, albeit its overall influence is not strictly contingent upon GPR109a signalling.

### 3.7 Ka Inhibits Osteoclast Differentiation and Function

**FIGURE 7:**
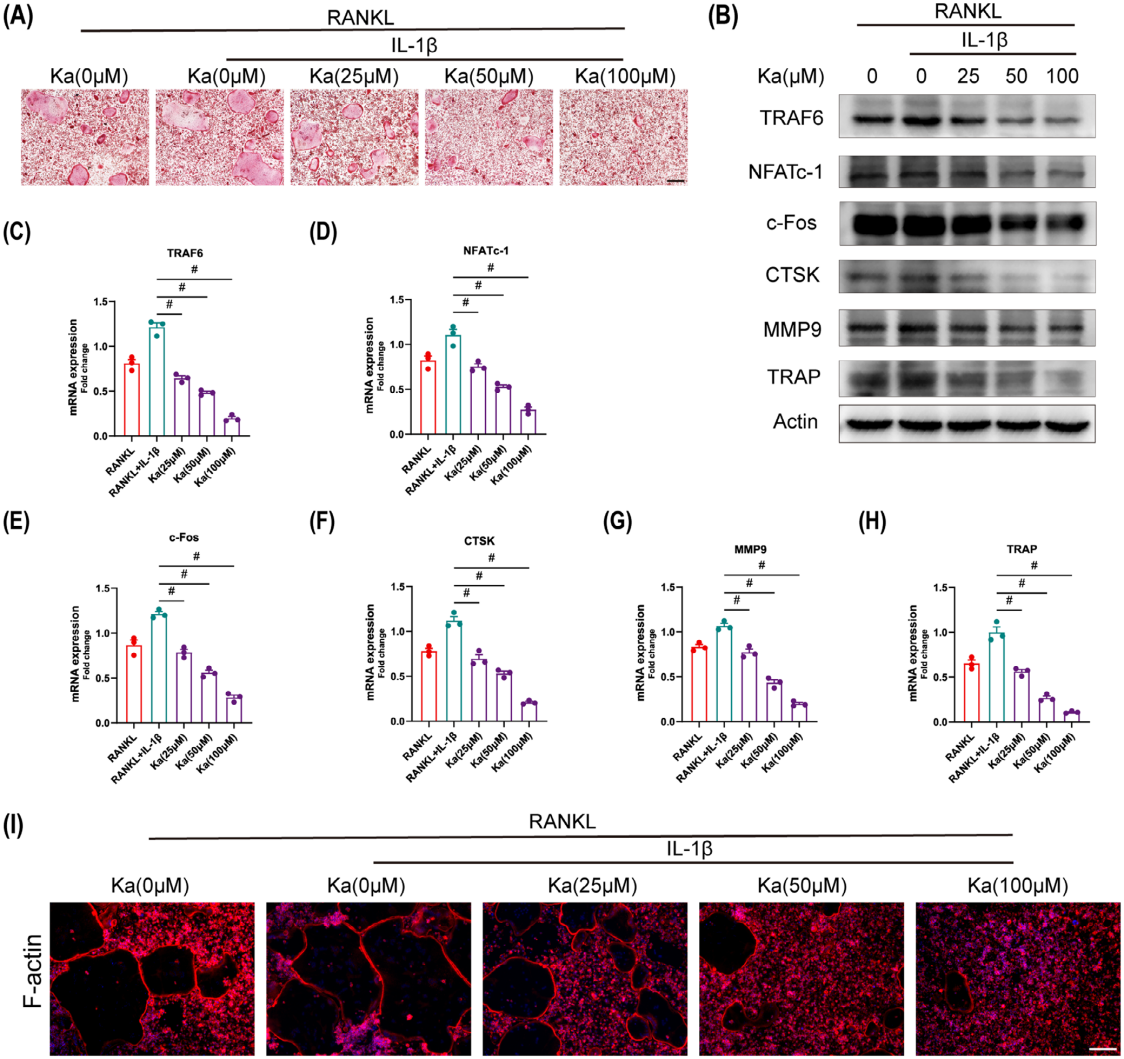
Ka inhibits osteoclast differentiation under IL‐1β stimulation. (A) General TRAP staining images of osteoclasts. (B) Immunoblotting analysis of protein expression related to osteoclast differentiation. (C–H) Related gene expression of mRNA in osteoclast differentiation. (I) Immunofluorescence analysis of F‐actin rings in osteoclasts. Scale bar was 200 μm. All data represent mean ± SEM. ^#^ *p* < 0.001.

Osteoclastogenesis, a key process in the progression of osteolysis, is intricately regulated by various factors [36]. Among these, IL‐1β, a key product of NLRP3 inflammasome activation, has been implicated as a promoter of osteoclast differentiation, exacerbating osteolytic processes [37]. Consequently, we sought to investigate whether Ka exerts a pivotal role in suppressing osteoclast differentiation under IL‐1β stimulation. To this end, we conducted in vitro studies by culturing BMDM with RANKL (50 ng/mL) and IL‐1β (20 ng/mL), either alone or in combination with Ka. Subsequent TRAP staining revealed that IL‐1β augmented osteoclast activity beyond that induced by RANKL alone, confirming its stimulatory effect on osteoclastogenesis (Figure 7A). Importantly, quantitative analysis of TRAP‐positive cells demonstrated that Ka, at varying concentrations, significantly reduced the number of multinucleated osteoclasts under IL‐1β stimulation, indicating a dosage‐responsive suppression of osteoclast maturation (Figure 7A).

**FIGURE 8:**
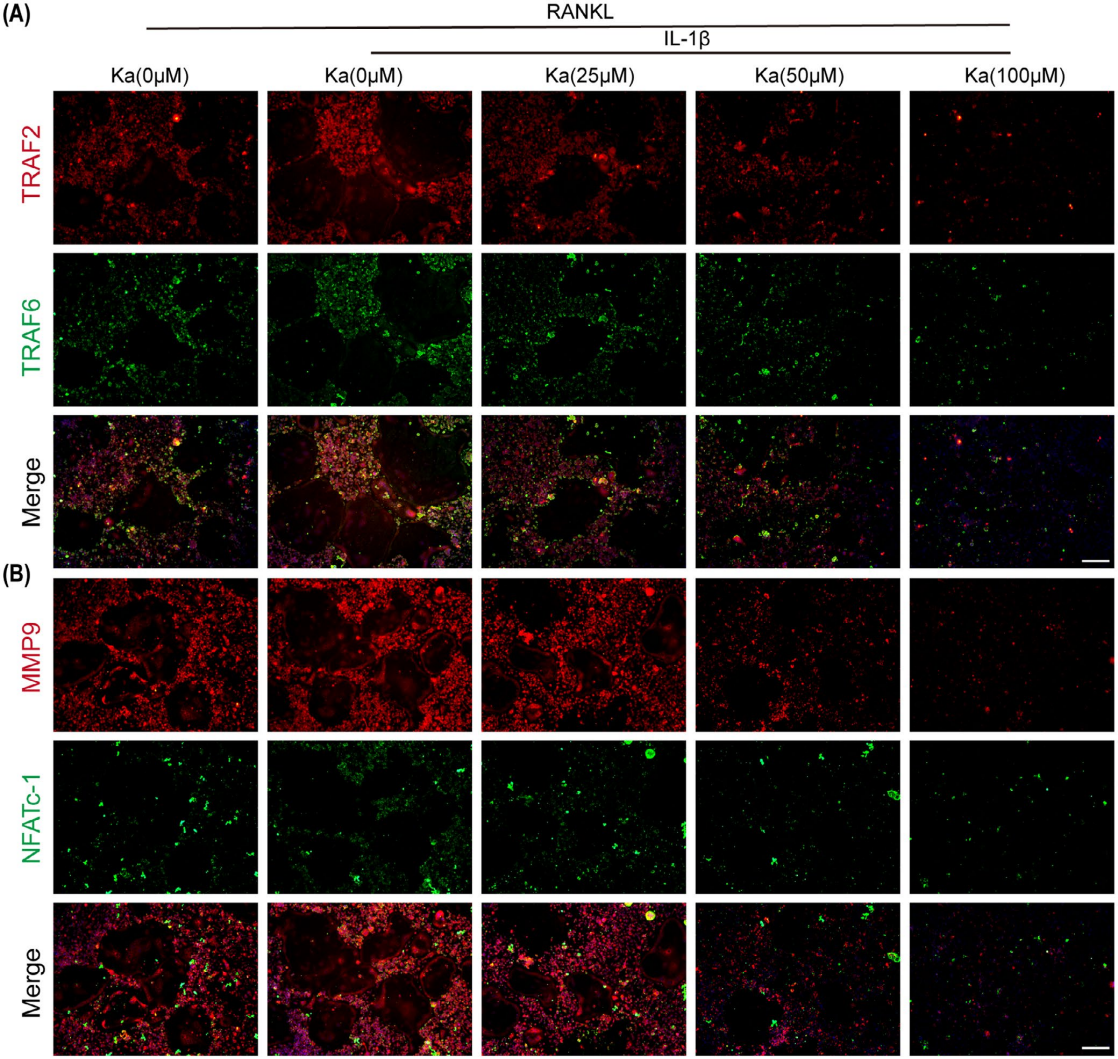
Ka inhibits RANKL‐induced osteoclast differentiation. (A) General immunofluorescence images of osteoclasts stained by TRAF2 (red) and TRAF6 (green). (B) General immunofluorescence images of osteoclasts stained by MMP9 (red) and NFATc‐1 (green). Scale bar was 200 μm.

To gain further insights into the molecular mechanisms underlying this inhibition, we employed immunoblotting to assess the expression of proteins crucial for osteoclastogenesis, such as NFATc‐1, TRAF6, c‐Fos, CTSK, MMP9 and TRAP. Our findings revealed that Ka exerted a suppressive effect on the expression levels of these proteins in a manner that corresponded to the dosage, throughout the process of osteoclast formation (Figures 7B and S5). Moreover, qRT‐PCR analysis of the corresponding gene expression corroborated our immunoblotting results, providing additional evidence for Ka's inhibitory effect at the transcriptional level (Figure 7C–H). We also examined the impact of Ka on the F‐actin ring structure, which is intimately linked to the bone resorptive capabilities of osteoclasts. As shown in Figure 7I, IL‐1β promoted the formation of F‐actin rings, whereas the administration of Ka resulted in a significant reduction in the quantity and dimensions of these rings, indicating an inhibitory effect on osteoclast function. This observation was further supported by immunofluorescence staining of TRAF2, TRAF6, MMP9 and NFATc‐1, which revealed similar trends (Figure 8).

**FIGURE 9:**
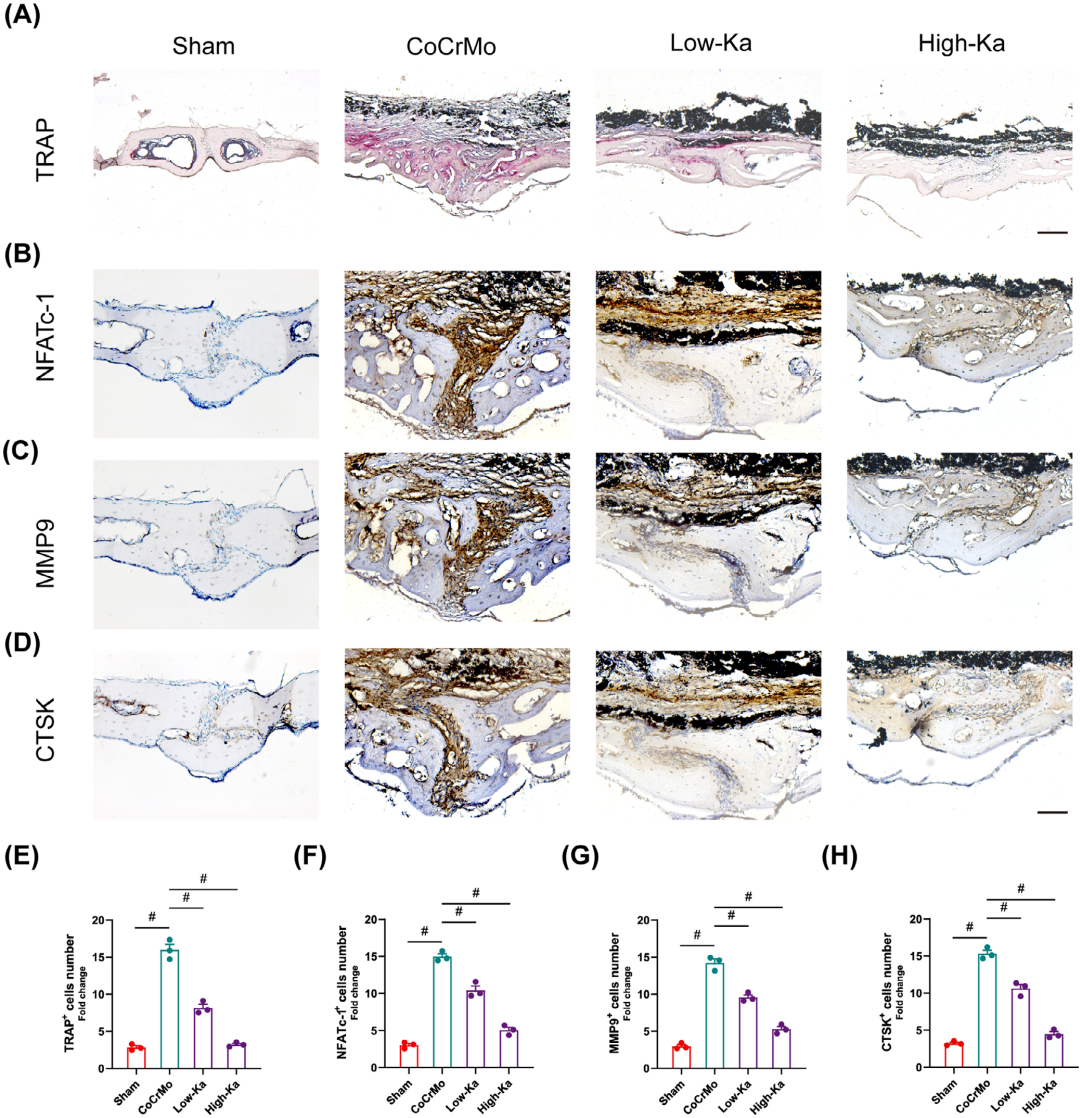
Ka inhibits osteoclast differentiation in vivo. (A) General images of calvarium slices stained by TRAP. Scale bar was 200 μm. (B–D) General images of immunohistochemical staining with (B) NFATc‐1, (C) MMP9 and (D) CTSK. Scale bar was 100 μm. (E–H) Positive cells quantification of (E) TRAP, (F) NFATc‐1, (G) MMP9 and (H) CTSK. All data represent mean ± SEM. ^#^ *p* < 0.001.

Subsequently, we conducted in vivo experiments to evaluate the impact of Ka on the process of bone resorption mediated by osteoclasts. As demonstrated by the TRAP staining of calvaria sections, a significant decrease in TRAP‐positive cells was observed following Ka administration (Figure 9A). Furthermore, we observed that Ka, at varying doses, effectively suppressed the expression of key proteins implicated in osteoclast differentiation and bone resorption, notably NFATc‐1, MMP9 and CTSK (Figure 9B–H). Collectively, these findings indicate that Ka exerts a mitigating effect on osteoclast differentiation, both in vivo and in vitro contexts.

### 3.8 Ka Promotes Osteogenic Differentiation Ability Under IL‐1β Stimulation

**FIGURE 10:**
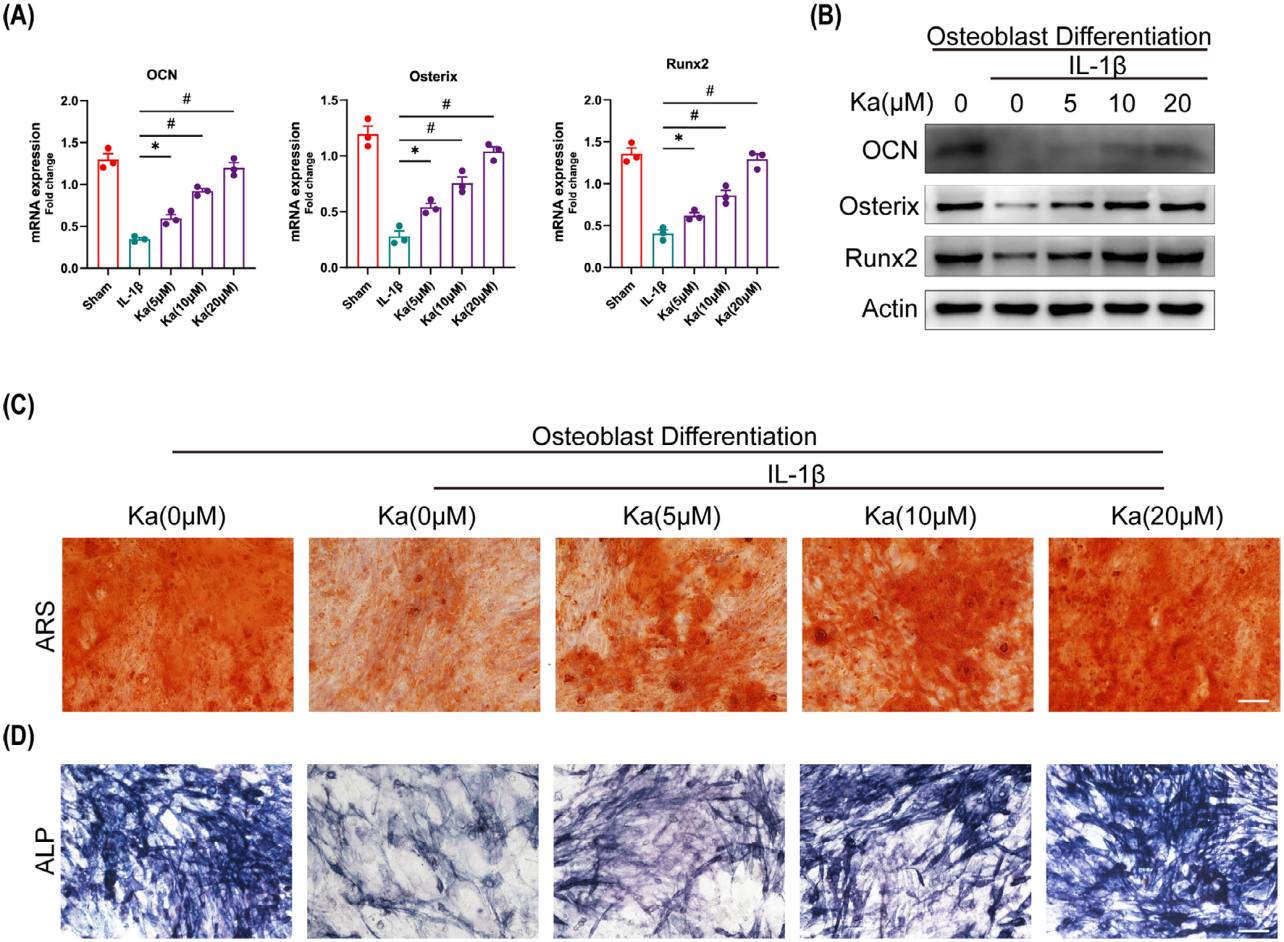
Ka accelerates osteogenic differentiation in vitro. (A) The expression of corresponding genes and (B) proteins in osteoblast differentiation. (C) General images of ARS and ALP staining in osteoblasts. Scale bar was 100 μm. All data represent mean ± SEM. *p < 0.05. ^#^ *p* < 0.001.

**FIGURE 11:**
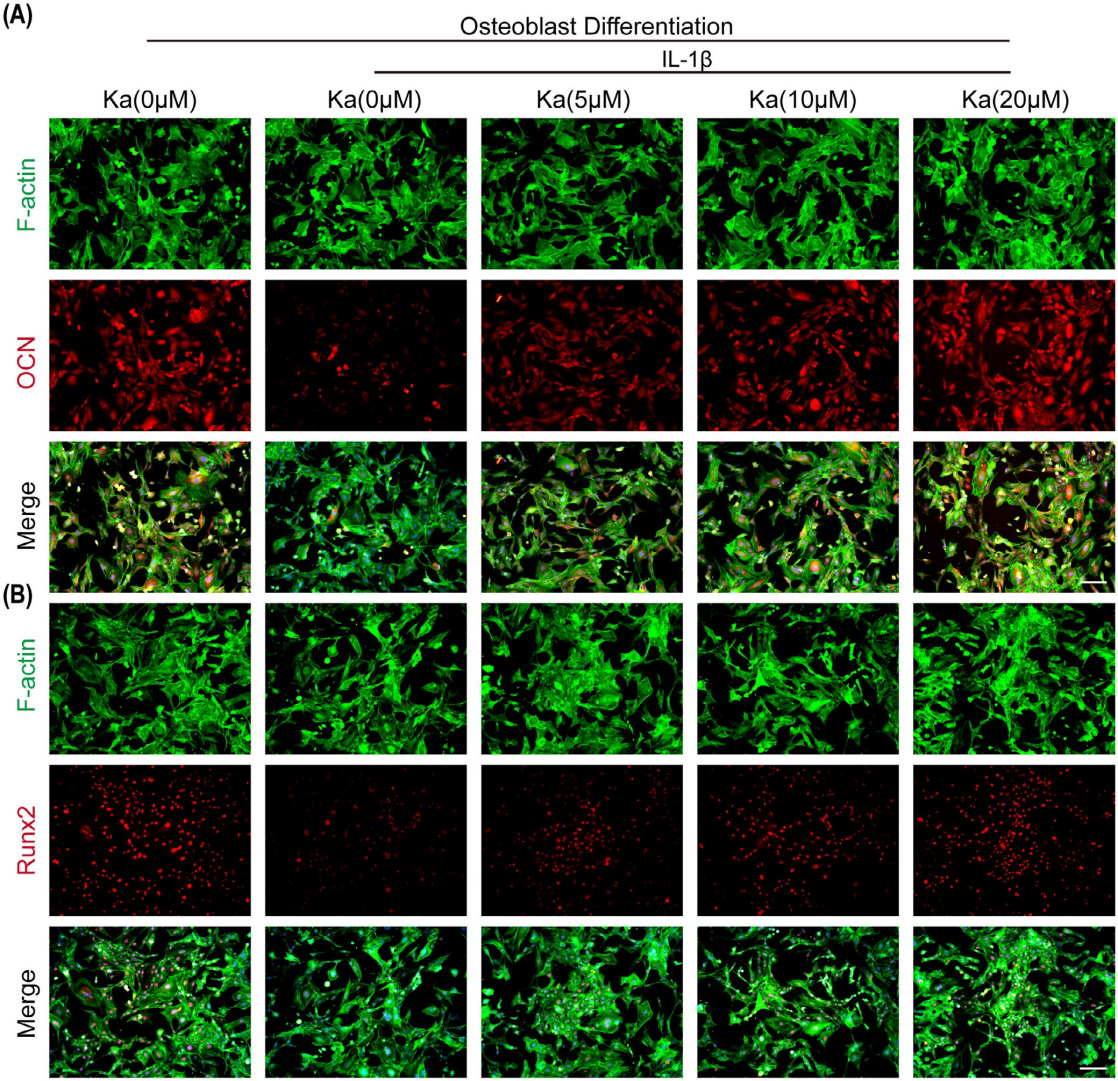
Ka promotes osteoblast differentiation. (A) General immunofluorescence images of osteoblasts stained by F‐actin and OCN. (B) General immunofluorescence images of osteoblasts stained by F‐actin and Runx2. Scale bar is 200 μm.

Since Ka has the ability to inhibit osteoclast differentiation, we further explored its possible involvement in osteogenic differentiation when influenced by IL‐1β. Preosteoblastic MC3T3‐E1 cells were subjected to co‐culturing with IL‐1β and varying concentrations of Ka in osteogenic medium. Following a seven‐day incubation period, cells were harvested using lysis buffer to quantify the expression patterns of osteogenic genes and proteins. As depicted in Figure 10A, the transcriptional levels of osteogenic differentiation‐related genes, including OCN, Osterix and Runx2, were markedly diminished upon exposure to IL‐1β. However, this inhibitory effect was notably reversed in the presence of Ka treatments. Notably, Ka also upregulated the expression of corresponding osteogenic proteins under IL‐1β stimulation, corroborating similar findings (Figure 10B). Subsequently, ARS and ALP staining assays were performed, confirming that Ka augmented ALP activity and mineralisation in IL‐1β‐treated cells (Figure 10C,D). To further substantiate these observations, immunofluorescence staining was conducted, revealing a dose‐dependent upregulation of osteoblast‐related proteins (OCN and Runx2) by Ka (Figure 11). Additionally, immunohistochemical staining of calvarium sections was undertaken to validate the effects of Ka on osteoblasts in vivo. The results indicated a significant enhancement in the expression of both OCN and Runx2 in groups administered with either low or high concentrations of Ka (Figures S6 and S7). Collectively, these findings underscore the ability of Ka to promote osteogenic differentiation.

## 4 Discussion

Total joint arthroplasty (TJA) is a cornerstone of orthopaedic surgery, offering patients relief from end‐stage arthritis and restoring joint function [38]. However, the long‐term success of TJA is frequently marred by periprosthetic osteolysis, a complication that results in aseptic loosening and ultimately culminates in prosthetic failure [31]. In the face of prosthesis failure, revision surgery stands as the sole viable therapeutic recourse, albeit it is fraught with significant drawbacks. Patients undergoing revision surgery often experience profound traumatic stress and pain, while the procedure itself may exacerbate disability and elevate mortality rates. Moreover, the financial implications of revision surgery are substantial, imposing a formidable burden on both individuals and healthcare systems [39]. The pathogenesis of this condition is multifactorial, with wear particles from the implant playing a central role in triggering an inflammatory cascade that disrupts bone homeostasis.

Our study addresses a critical gap in the literature by exploring the role of Ka, a bioactive flavonoid, in modulating the NLRP3 inflammasome and its downstream effects on bone metabolism. The NLRP3 inflammasome, a crucial multiprotein complex that orchestrates the maturation and release of pro‐inflammatory cytokines, has been identified as a key mediator in the development of osteolysis triggered by wear particles [40, 41]. Specifically, wear debris from implants activates the NLRP3 inflammasome, initiating a cascade of events that ultimately leads to pyroptotic cell death. This intricate process involves the activation of caspase‐1, which subsequently triggers the maturation of IL‐1β. Concurrently, caspase‐1 induces the cleavage of GSDMD, leading to the generation of its N‐terminal fragment, GSDMD‐N. This GSDMD‐N fragment is crucial as it mediates the disruption of the cell membrane, enabling the secretion of mature IL‐1β and ultimately leading to pyroptotic cell demise [34]. This elucidation of the underlying mechanism offers a novel and promising new pathway for developing targeted therapeutic strategies aimed at mitigating osteolysis.

Flavonoids are natural polyphenols enriched in vegetables and fruits with a plethora of physiological functions including antitumor, anti‐inflammatory, antibacterial, antioxidant, osteogenic and anti‐osteoclastogenic effects [23]. Among these flavonoids, Ka has garnered attention for its potential therapeutic role in various inflammatory disorders [22, 24]. Recently, Ka has demonstrated neuroprotective effects in mice by inhibiting NLRP3 inflammasome activation, thereby mitigating neurodegeneration [42]. Furthermore, kaempferide, a structural analog of Ka, has exhibited efficacy in preventing wear particle–induced bone resorption [43]. However, the direct impact of Ka on osteolysis has remained unexplored until now.

By targeting the NLRP3 inflammasome, we hypothesised that Ka could offer a novel therapeutic approach to mitigate osteolysis and enhance the longevity of TJA. Within the scope of this research, we systematically investigated the effect of Ka on osteolysis induced by CoCrMo alloy particles and found that Ka significantly inhibited this process in a dose‐dependent manner. Subsequently, we delved into the underlying mechanisms responsible for Ka's inhibitory effect on osteolysis. Although the capacity of Ka to inhibit NLRP3 inflammasome activation is well established, its function in controlling the inflammatory response to wear particles has remained elusive. Our findings demonstrated that Ka effectively alleviates NLRP3 inflammasome activation triggered by wear particles.

Moreover, we discovered that Ka inhibits pyroptosis by regulating ASC oligomerisation and GSDMD cleavage, providing further insight into its mechanistic pathways. Furthermore, our investigation aimed to ascertain whether Ka's inhibitory impact on NLRP3 inflammasome activation is exerted via the GPR109a receptor. Prior research has suggested that this receptor contributes to the modulation of the NLRP3 inflammasome [35]. Our results indicate that while Ka activates GPR109a, its suppressive effect on the NLRP3 inflammasome is not solely dependent on this receptor, suggesting a more complex mode of action.

Bone metabolism, an intricate process involving the coordinated activities of osteoblasts and osteoclasts, is characterised by a dynamic equilibrium between bone formation and resorption [44, 45]. The delicate homeostasis maintained between osteoclastic bone resorption and osteoblastic bone regeneration is paramount for preserving the structural integrity of bone tissue. Among the plethora of factors influencing this process, IL‐1β, a pivotal product of pyroptosis, has garnered significant attention for its profound effects on bone metabolism, as evidenced by numerous studies [19, 37, 46]. Furthermore, flavonoids have garnered attention for their ability to modulate the functions of osteoblasts and osteoclasts, thereby maintaining the metabolic balance during bone remodelling [19, 47]. Ka, a specific polyphenolic flavonoid, has been shown to exert dual effects, inhibiting osteoclast formation [46] while promoting osteoblast differentiation [48]. However, the persistence of these effects in an inflammatory milieu characterised by IL‐1β remains elusive, necessitating further investigation.

To address this gap, we conducted a study examining the impact of Ka on osteoclastogenesis and osteoblast differentiation in an inflammatory environment enriched with IL‐1β. Our findings revealed that Ka markedly suppressed the differentiation and formation of osteoclasts, even under IL‐1β stimulation, through the downregulation of relevant genes and proteins. Additionally, Ka has been demonstrated to upregulate the expression of key osteogenic markers, including Runx2, Osterix and OCN, leading to increased activity of ALP and enhanced mineralisation in osteoblasts. Collectively, these findings indicate that Ka is crucial for preserving the balance between osteoclastic bone resorption and osteoblastic bone formation by modulating the differentiation of both cell types.

The ability of Ka to modulate both inflammatory and metabolic dimensions of bone pathology highlights its promise as a therapeutic agent with dual mechanisms of action. Its anti‐inflammatory properties are particularly notable, as evidenced by the suppression of NLRP3 inflammasome activation, which consequently leads to a marked decrease in pyroptotic cell death. Concurrently, its promotion of osteogenic differentiation and suppression of osteoclastogenesis highlight its anabolic potential in maintaining bone integrity. The implications of our findings extend beyond the management of periprosthetic osteolysis. Ka's multifaceted mechanisms of action suggest that it could be an asset in the broader context of bone disease management, including osteoporosis and periodontitis, where inflammation and dysregulated bone metabolism are also critical factors.

In conclusion, our study reveals the therapeutic potential of Ka in mitigating osteolysis triggered by wear particles by the modulation of the NLRP3 inflammasome and the restoration of bone metabolic balance. Collectively, these findings lay the groundwork for future investigations into the role of natural polyphenols in orthopaedic medicine and open new avenues for the advancement of targeted therapies for bone diseases.

## Author Contributions

**Cheng Huang:** investigation (lead), methodology (lead), writing – original draft (lead). **Chenhui Zhang:** investigation (equal), methodology (equal), writing – original draft (equal). **Yongjun Luo:** investigation (equal), methodology (equal). **Lujun Guo:** data curation (equal). **Yanglin Wu:** data curation (equal), software (equal). **Qingyan Shi:** data curation (equal). **Yazhong Zhang:** conceptualization (equal), project administration (equal), supervision (equal). **Chengyuan Yang:** conceptualization (equal), project administration (equal), supervision (equal), writing – original draft (equal). **Bo Wang:** conceptualization (equal), project administration (equal), supervision (equal). **Junjie Niu:** conceptualization (lead), project administration (lead), supervision (lead), writing – review and editing (lead). **Jun Lin:** writing – review and editing (equal).

## Disclosure

Author Agreement: All authors have seen and approved the final version of the manuscript being submitted. They warrant that the article is the authors' original work, has not received prior publication and is not under consideration for publication elsewhere.

## Ethics Statement

This study was conducted with the approval of the Ethics Committee of Soochow University (SUDA20221108A03).

## Conflicts of Interest

The authors declare no conflicts of interest.

## Supporting information


**Table S1:** Primer sequences used in the qRT‐PCR analysis.
**Figure S1:** The characteristic of CoCrMo alloy particles. (A) Representative scanning electron microscopy (SEM) image of CoCrMo alloy particles. The scale bar was 200 nm.
**Figure S2:** Ka inhibits NLRP inflammasome activation. LPS‐primed BMDMs were incubated with Ka and then stimulated with CoCrMo particles. (A) The cell lysates were detected for NLRP3 by western blot. (B) Quantification of NLRP3. All data represent mean ± SEM. ^#^ *p* < 0.001.
**Figure S3:** Ka inhibits NLRP3 inflammasome activation in vivo. (A–C) Immunohistochemical staining of (A) NLRP3, (B) Caspase‐1 and (C) IL‐1β. Scale bar: 100 μm. (D–F) Quantification of (D) NLRP3 positive cells, (E) Caspase‐1 positive cells and (F) IL‐1β positive cells. *n* = 3. All data represent mean ± SEM. ^#^ *p* < 0.001.
**Figure S4:** The effect of Ka on osteolysis partially depended on the GPR109a receptor. (A) immunohistochemical staining of NLRP3, (B) Caspase‐1 and (C) IL‐1β. The scale bar was 100 μm. (D) Quantification of NLRP3 positive cells, (E) Caspase‐1 positive cells and (F) IL‐1β positive cells. *n* = 3. All data represent mean ± SEM. ^#^ *p* < 0.001.
**Figure S5:** Ka inhibited osteoclast differentiation in vitro. Quantitation of TRAF6, NFATc‐1, c‐Fos, Trap, MMP9 and CTSK band intensity as fold change. All data represent mean ± SEM. ^#^ *p* < 0.001.
**Figure S6:** Ka promotes the expression of osteogenic markers in vivo. (A‐B) Immunohistochemical staining of (A) OCN and (B) Runx2. The scale bar was 100 μm. (C‐D) Quantification of (C) OCN positive cells and (D) Runx2 positive cells. *n* = 3. All data represent mean ± SEM. **p < 0.01. ^#^ *p* < 0.001.
**Figure S7:** Ka inhibited osteoblast differentiation in vitro. Quantitation of OCN, osterix and Runx2 band intensity as fold change. All data represent mean ± SEM. *p < 0.05. ^#^ *p* < 0.001.

## Data Availability

The Western blot, immunohistochemistry and other related experimental data generated and analysed during the current study are included within this published article and its Data S1. Additional data are available from the corresponding author upon reasonable request.
