# Supplementary material for: The Flavonoid Kaempferol Mitigates Periprosthetic Osteolysis by Regulating the NLRP3 Inflammasome and Balancing Bone Metabolism
Source: J Cell Mol Med. 2025 Oct 6;29(19):e70878. doi: 10.1111/jcmm.70878 (PMC12500419; doi:10.1111/jcmm.70878)
Supplement: Supplementary file 1 — Table S1: Primer sequences used in the qRT‐PCR analysis. Figure S1: The characteristic of CoCrMo alloy particles. (A) Representative scanning electron microscopy (SEM) image of CoCrMo alloy particles. The scale bar was 200 nm. Figure S2: Ka inhibits NLRP inflammasome activation. LPS‐primed BMDMs were incubated with Ka and then stimulated with CoCrMo particles. (A) The cell lysates were detected for NLRP3 by western blot. (B) Quantification of NLRP3. All data represent mean ± SEM. # p < 0.001. Figure S3: Ka inhibits NLRP3 inflammasome activation in vivo. (A–C) Immunohistochemical staining of (A) NLRP3, (B) Caspase‐1 and (C) IL‐1β. Scale bar: 100 μm. (D–F) Quantification of (D) NLRP3 positive cells, (E) Caspase‐1 positive cells and (F) IL‐1β positive cells. n = 3. All data represent mean ± SEM. # p < 0.001. Figure S4: The effect of Ka on osteolysis partially depended on the GPR109a receptor. (A) immunohistochemical staining of NLRP3, (B) Caspase‐1 and (C) IL‐1β. The scale bar was 100 μm. (D) Quantification of NLRP3 positive cells, (E) Caspase‐1 positive cells and (F) IL‐1β positive cells. n = 3. All data represent mean ± SEM. # p < 0.001. Figure S5: Ka inhibited osteoclast differentiation in vitro. Quantitation of TRAF6, NFATc‐1, c‐Fos, Trap, MMP9 and CTSK band intensity as fold change. All data represent mean ± SEM. # p < 0.001. Figure S6: Ka promotes the expression of osteogenic markers in vivo. (A‐B) Immunohistochemical staining of (A) OCN and (B) Runx2. The scale bar was 100 μm. (C‐D) Quantification of (C) OCN positive cells and (D) Runx2 positive cells. n = 3. All data represent mean ± SEM. **p < 0.01. # p < 0.001. Figure S7: Ka inhibited osteoblast differentiation in vitro. Quantitation of OCN, osterix and Runx2 band intensity as fold change. All data represent mean ± SEM. *p < 0.05. # p < 0.001. [file JCMM-29-e70878-s001.docx]

Supplementary Information

**The Flavonoid** **Kaempferol Mitigates Periprosthetic Osteolysis by Regulating NLRP3 Inflammasome and Balancing Bone Metabolism**

Cheng Huang^1#^, Chenhui Zhang^2#^, Yongjun Luo^2#^, Lujun Guo^2^, Yanglin Wu^2^, Qingyan Shi^2^, Yazhong Zhang^3*^, Chengyuan Yang^4*^, Bo Wang^5*^, Junjie Niu^4*^, Jun Lin^2^

^1^ Department of Orthopeadics, China-Japan Friendship Hospital, Beijing 100029, China

^2^ Department of Orthopaedics, The Fourth Affiliated of Soochow University, SuzhouDushu Lake Hospital, Medical Center of Soochow University, Suzhou 215000, China

^3^ Department of Orthopaedics, The Second Affiliated Hospital of XuZhou Medical University，Xuzhou 221006, China

^4^ Department of Orthopaedics, The First Affiliated Hospital of Soochow University, Soochow University, Suzhou 215006, China

^5^ Department of Spinal Surgery, Shanghai East Hospital, School of Medicine, Tongji University, Shanghai 200092, China

^#^ These authors contributed equally.

**Corresponding Authors:**

Yazhong Zhang, Department of Orthopaedics, The Second Affiliated Hospital of XuZhou Medical University，Xuzhou 221006, China. Email: zyz930507@163.com

Chengyuan Yang, Department of Orthopaedics, The First Affiliated Hospital of Soochow University, Soochow University, Suzhou 215006, China. Email: ycy8991@126.com

Bo Wang, Department of Spinal Surgery, Shanghai East Hospital, School of Medicine, Tongji University, Shanghai 200092, China. Email: Drwangbo0216@126.com

Junjie Niu, Department of Orthopaedics, The First Affiliated Hospital of Soochow University, Soochow University, Suzhou 215006, China. Email: niujunjie1129@suda.edu.cn

1. **Experimental procedures**
   1. **Micro-CT analysis**

Fixed calvarium were analyzed by a high-resolution micro-CT (micro-computed tomography) (SkyScan 1176, Belgium). Before scanning, the CoCrMo particles on the calvarium were eliminated to avoid metal artifacts. Then, all samples were assessed at 9 μm, 50 kV, and 500 μA. After 3D image rebuilding, a region of interest (ROI) around the sagittal suture was chosen for the following quantitative analysis. Morphometric parameters including Bone Mineral Density (BMD), Bone Volume/Total Volume (BV/TV), and total porosity were measured by using the SkyScan reconstruction program.

**1.2 Cell cultures**

BMDMs and THP-1 cells: BMDMs were collected from the tibia and femur of C57BL/6 mice. In brief, the sacrificed mice were first soaked in 75% ethanol for 2 min for sterilization. Then, the tibia and femur were separated and flushed with a complete DMEM medium after the soft tissues were cleaned out. Next, the medium with bone marrow was centrifuged for 5 min before the erythrocytes were removed by red blood cell lysis buffer. Finally, cells were cultured in DMEM with 10% FBS and 40 ng/ml M-CSF for 5-7 days. THP-1 cells purchased from Procell (Wuhan, China) were maintained in RPMI 1640 medium supplemented with 10% FBS and 100 units/ml Penicillin–Streptomycin in 5% CO_2_ at 37℃. Then, cells were differentiated into macrophages by incubation with 100 ng/ml PMA for 3 h. Mature BMDMs and differentiated THP-1 macrophages were incubated with LPS for 3 hours before being stimulated by CoCrMo alloy particles at a concentration of 0.1 mg/ml and treated with different doses of Ka. Cell lysate and supernatant were evaluated by Western Blot and ELISA.

Osteoclast: Total BMMs were isolated from the tibia and femur of C57BL/6 mice. The cells were maintained in a 100 mm dish in a complete α-MEM medium at 37°C and 5% CO_2_ overnight, and then further cultured in 24-well plates with α-MEM medium containing 40 ng/ml M-CSF. The next day, 50 ng/ml RANKL and different doses of Ka were supplemented in these plates. The osteoclast medium was replaced every 2 days. After 5-7 days, differentiated osteoclasts were used for further study.

Osteoblast: MC3T3-E1 cells were cultured in a complete α-MEM medium with 10% FBS (fetal bovine serum) and 1% antibiotic. For osteoblast differentiation, 0.1 μM dexamethasone, 0.5 mM vitamin C, and 10 mM β-glycerophosphate were supplemented into the α-MEM medium. Then cells were treated with or without IL-1β (20 ng/ml) before being used for further study. The osteoblast medium was replaced every 3 days.

**1.3 Analysis of ASC oligomerization**

NLRP3 inflammasome activation was performed in BMDMs as mentioned above. After being treated as desired, cells were washed with 500 μM PBS and then lysed in a cold lysis butter supplemented with 1 mM PMSF (phenylmethylsulfonyl fluoride) and 0.5% Triton X-100 for 30 min. Next, cells were scraped, and the lysates were centrifuged at 3000 g for 10 min at 4 ℃. After two washes in cold PBS, the insoluble pellets were resuspended in 200 μL cold PBS butter containing 2 mM DSS (disuccinimidyl suberate) and incubated for 30 min at room temperature with rotation. Finally, the cross-linked samples were resuspended in 1X SDS loading butter after centrifugation and then boiled at 100 ℃ for 5 min before being analyzed by Western Blot.

For the analysis of ASC speck formation, BMDMs were seeded into a 24-well plate and then treated as desired. After treatment, cells were fixed with 4% paraformaldehyde for 10 min before being blocked by blocking butter. Next, a primary antibody anti-ASC was used for the primary incubation at 4 ℃ overnight. The next day, cells were incubated with the fluorescent secondary antibody at 24 ℃ for one hour before being incubated with DAPI. The ASC specks were observed by an inverted fluorescence microscope.

- 1. **qRT-PCR**

Total RNAs were extracted from osteoclasts and osteoblasts with a Simply P Total RNA Extraction Kit under the guidance of the manufacturer’s instruction. The concentration of RNA was assessed by a NanoDrop-2000 spectrophotometer. The generation of cDNA and Q-PCR were performed according to the manufacturer’s protocol. All PCR primers were listed in Table S1.

| Gene | Forward | Reverse |
| --- | --- | --- |
| *TRAF6* | 5’AAAGCGAGAGATTCTTTCCCTG3’ | 5’ACTGGGGACAATTCACTAGAGC3’ |
| *NFATc-1* | 5’GGTGCCTTTTGCGAGCAGTATC3’ | 5’CGTATGGACCAGAATGTGACGG3’ |
| *Trap* | 5’CGACCATTGTTAGCCACATACG3’ | 5’TCGTCCTGAAGATACTGCAGGTT3’ |
| *Ctsk* | 5’AGGGCCAACTCAAGAAGAAAACT3’ | 5’TGCCATAGCCCACCACCAACACT3’ |
| *Mmp9* | 5’GCTGACTACGATAAGGACGGCA3’ | 5’TAGTGGTGCAGGCAGAGTAGGA3’ |
| *OCN* | 5’TTGAACTGTTTGTTTTGGACCC3’ | 5’CCAACAGACACCAGTTGTAAAG3 |
| *Osterix* | 5’TGAGCTGGAACGTCACGTGC3’ | 5’AAGAGGAGGCCAGCCAGACA3 |
| *Runx2* | 5’TTGACCTTTGTCCCAATGC3’ | 5’AGGTTGGAGGCACACATAGG3’ |

**Table S1.** Primer sequences used in the qRT-PCR analysis.

- 1. **Western blotting**

The cellular homogenization process was initiated using a lysis buffer enriched with protein inhibitors. Subsequently, the protein concentration within the cell lysate was accurately determined utilizing a BCA (bicinchoninic acid) assay kit. Following this quantification step, the proteins were separated by SDS-PAGE under carefully controlled conditions: an initial voltage of 80 V was applied for 25 minutes, followed by an increase to 120 V for an additional 60 minutes. The subsequent transfer of proteins to PVDF (polyvinylidene fluoride) membranes was performed under a constant current of 350 mA for a duration of one hour. Prior to antibody incubation, the membranes were blocked with Quickblock buffer for 30 minutes to minimize non-specific binding. Subsequently, the membranes were incubated with the primary antibodies overnight under conditions that facilitate optimal antibody binding to the target proteins. Following three thorough washes to remove unbound antibodies, the membranes were incubated with secondary antibodies for one hour at 24°C, facilitating the detection of the primary antibody-protein complexes. Finally, the relative abundance of these proteins was quantified using the ImageJ software package.

**
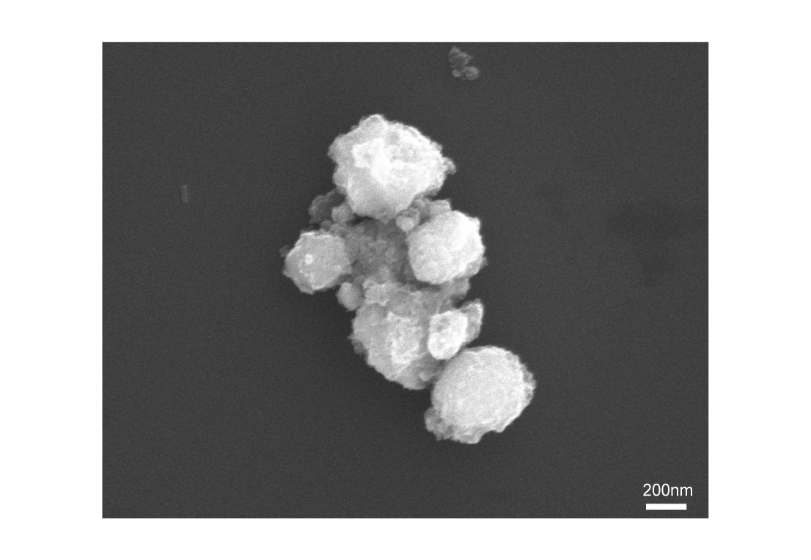
**

**Figure S1.** The characteristic of CoCrMo alloy particles. **(A)** Representative scanning electron microscopy (SEM) image of CoCrMo alloy particles. The scale bar was 200 nm.


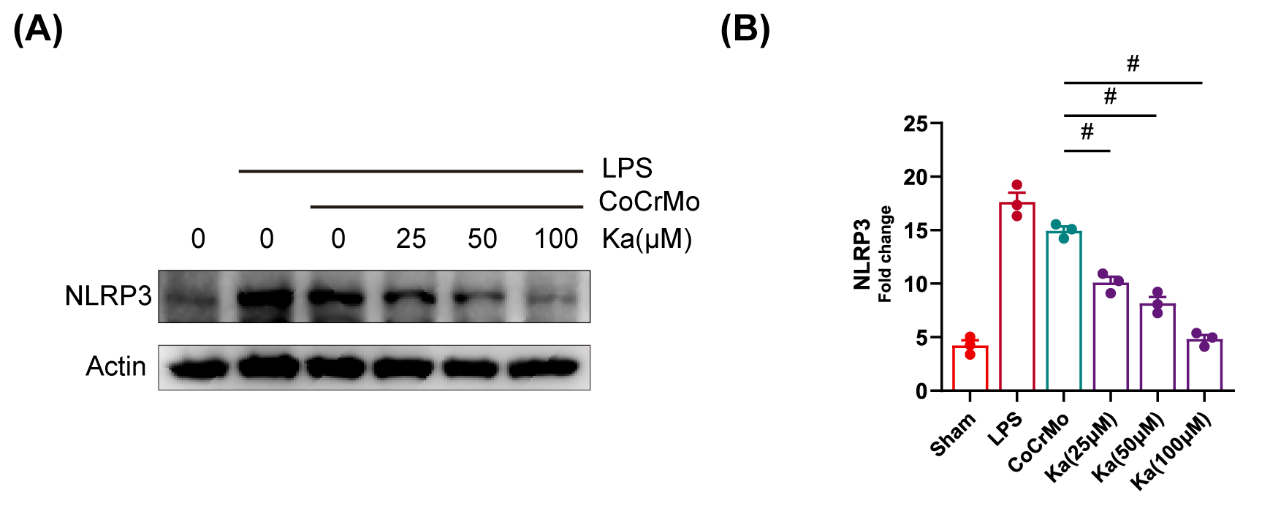


**Figure S2.** Ka inhibits NLRP inflammasome activation. LPS-primed BMDMs were incubated with Ka and then stimulated with CoCrMo particles. **(A)** The cell lysates were detected for NLRP3 by western blot. **(B)** Quantification of NLRP3. All data represent mean ± SEM. ^#^ p < 0.001.


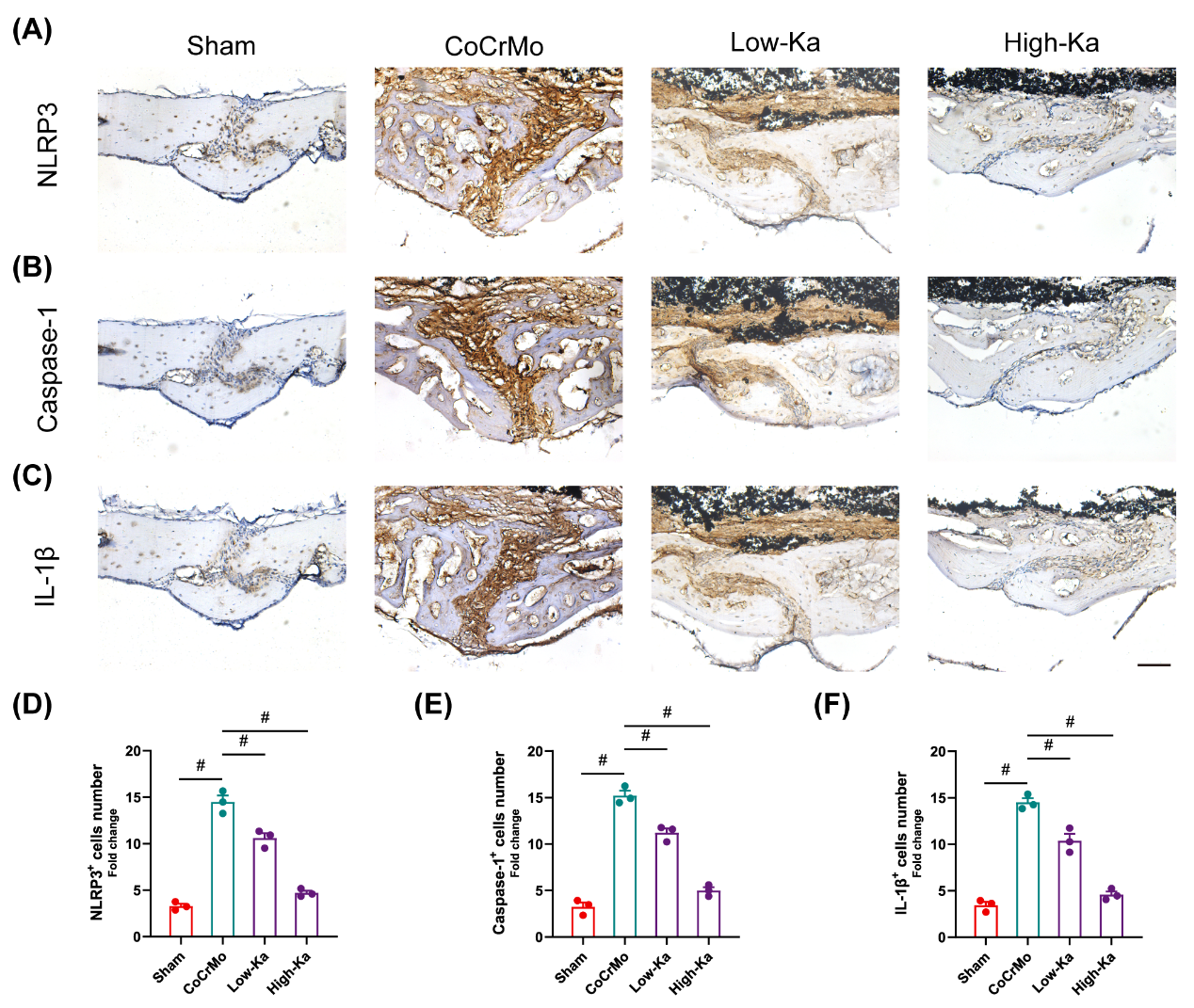


**Figure S3.** Ka inhibits NLRP3 inflammasome activation in vivo. **(A-C)** Immunohistochemical staining of **(A)** NLRP3, **(B)** Caspase-1, and **(C)** IL-1β. Scale bar: 100μm. **(D-F)** Quantification of **(D)** NLRP3 positive cells, **(E)** Caspase-1 positive cells, and **(F)** IL-1β positive cells. n=3. All data represent mean ± SEM. ^#^ p < 0.001.


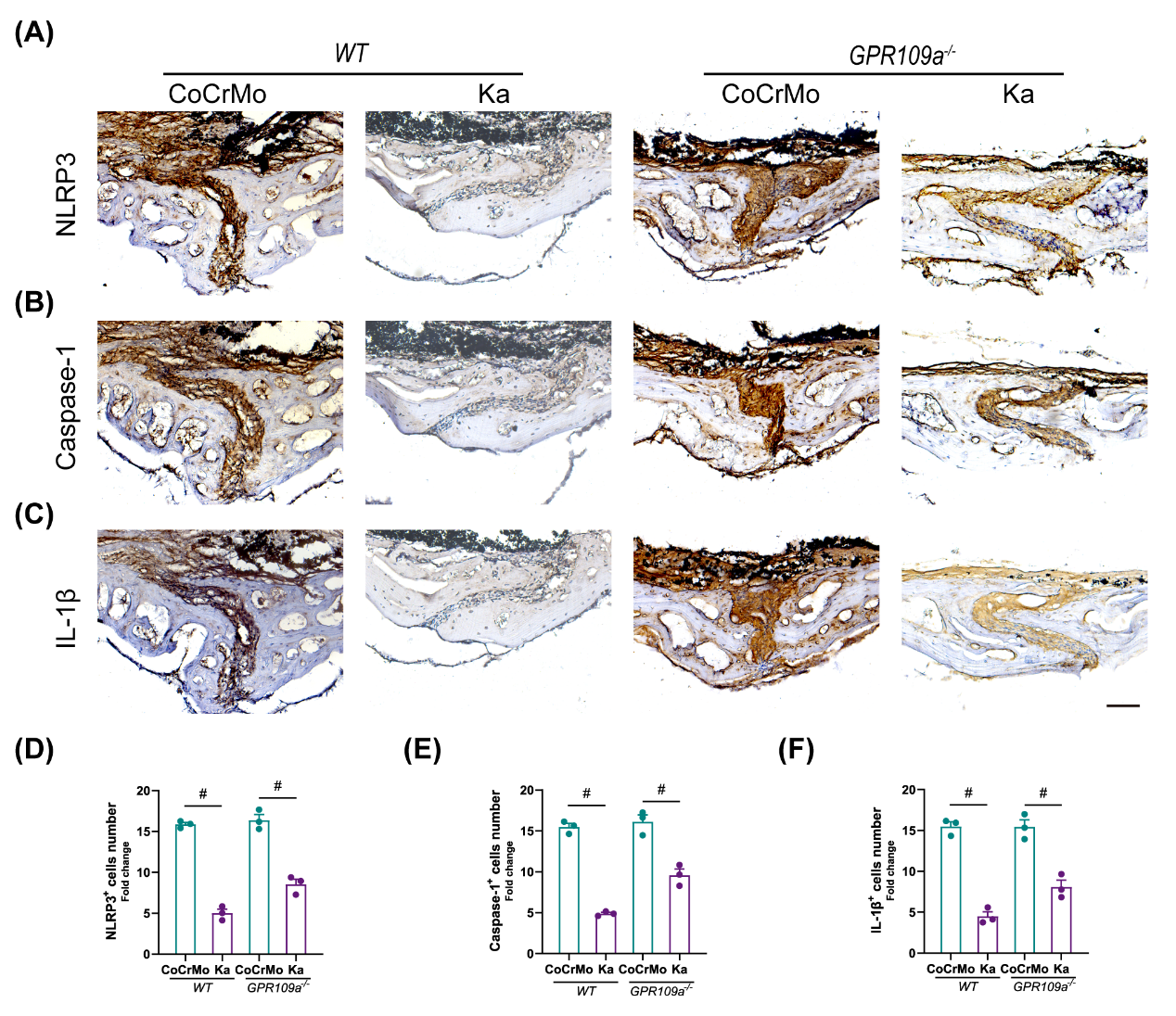


**Figure S4.** The effect of Ka on osteolysis partially depended on the GPR109a receptor. **(A)** immunohistochemical staining of NLRP3, **(B)** Caspase-1 and **(C)** IL-1β. The scale bar was 100 μm. **(D)** Quantification of NLRP3 positive cells, **(E)** Caspase-1 positive cells, and **(F)** IL-1β positive cells. n=3. All data represent mean ± SEM. ^#^ p < 0.001.

**
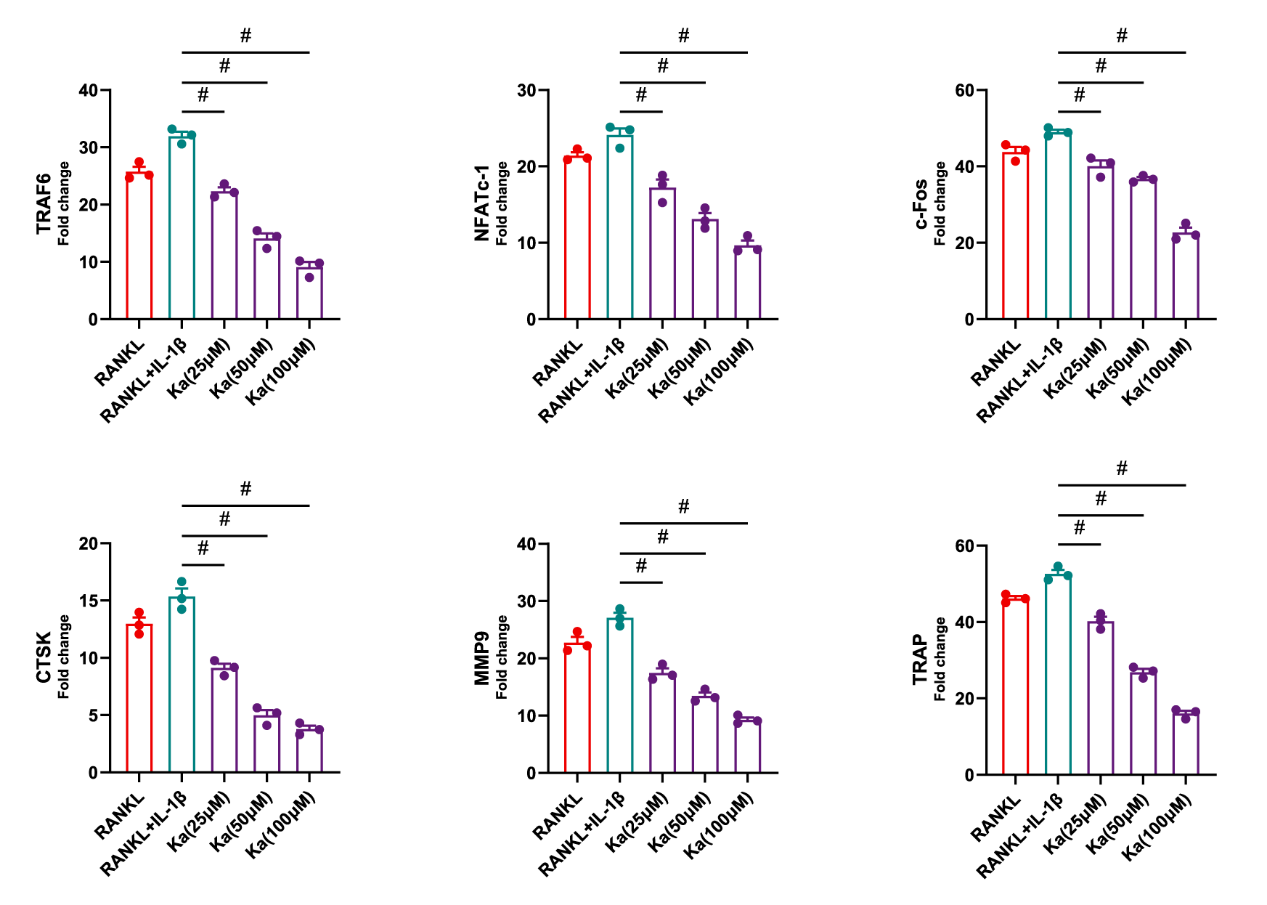
**

**Figure S5.** Ka inhibited osteoclast differentiation in vitro. Quantitation of TRAF6, NFATc-1, c-Fos, Trap, MMP9, and CTSK band intensity as fold change. All data represent mean ± SEM. ^#^ p < 0.001.

**
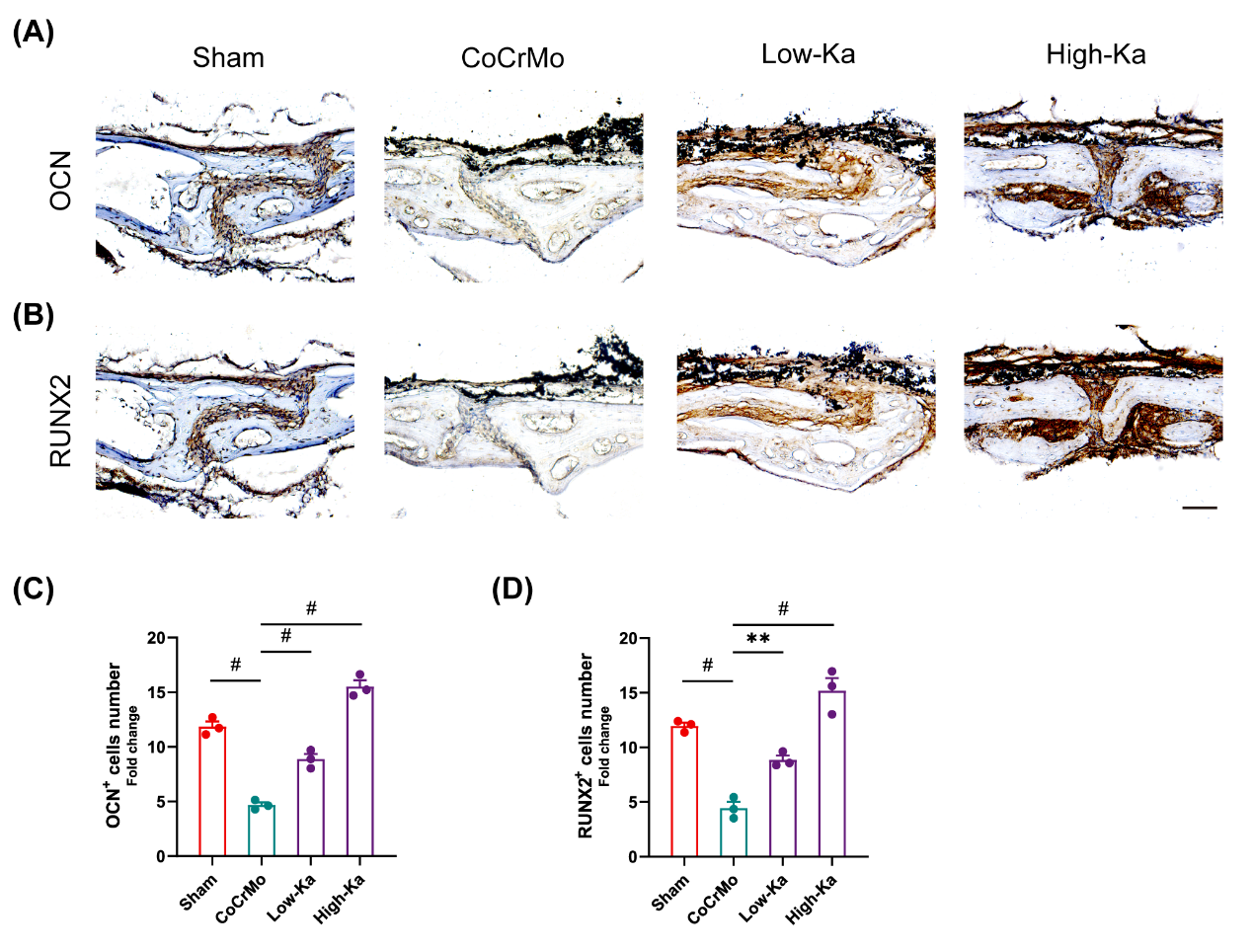
**

**Figure S6.** Ka promotes the expression of osteogenic markers in vivo. **(A-B)** Immunohistochemical staining of **(A)** OCN and **(B)** Runx2. The scale bar was 100μm. **(C-D)** Quantification of **(C)** OCN positive cells and **(D)** Runx2 positive cells. n=3. All data represent mean ± SEM. ** p < 0.01. ^#^ p < 0.001.


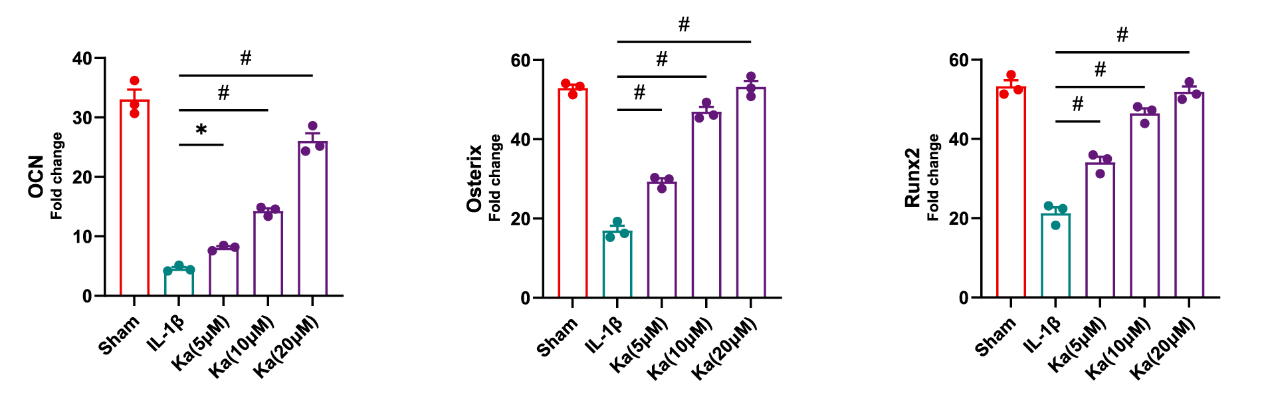


**Figure S7.** Ka inhibited osteoblast differentiation in vitro. Quantitation of OCN, osterix, and Runx2 band intensity as fold change. All data represent mean ± SEM. * p < 0.05. ^#^ p < 0.001.
